# Expression profile-based screening for critical genes reveals S100A4, ACKR3 and CDH1 in docetaxel-resistant prostate cancer cells

**DOI:** 10.18632/aging.102600

**Published:** 2019-12-29

**Authors:** Sha Zhu, Zhixue Min, Xianli Qiao, Shengxian Chen, Jian Yang, Xiao Zhang, Xigang Liu, Weijie Ran, Renguang Lv, Ying Lin, Jin Wang

**Affiliations:** 1 Key laboratory of Tumor Immunology, Center of Infection and Immunization, Department of Immunology, College of Basic Medical Sciences, Zhengzhou University, Zhengzhou 450001, P. R. China; 2 The Third People’s Hospital of Zhengzhou, Zhengzhou 450000, P.R. China; 3 School of Medicine, Shanghai Jiao Tong University, Shanghai 20040, P.R. China; 4 Collaborative Innovation Center of Cancer Chemoprevention, School of Physics and Engineering, Zhengzhou University, Zhengzhou 450001, P. R. China

**Keywords:** differentially expressed genes, microarray, docetaxel, AIPC

## Abstract

Docetaxel is a first-line anticancer drug widely used in the treatment of advanced prostate cancer. However, its therapeutic efficacy is limited by its side effects and the development of chemoresistance by the tumor. Using a gene differential expression microarray, we identified 449 genes differentially expressed in docetaxel-resistant DU145 and PC3 cell lines as compared to docetaxel-sensitive controls. Moreover, western blotting and immunohistochemistry revealed altered expression of S100A4, ACKR3 and CDH1in clinical tumor samples. Cytoscape software was used to investigate the relationship between critical proteins and their signaling transduction networks. Functional and pathway enrichment analyses revealed that these signaling pathways were closely related to cellular proliferation, cell adhesion, cell migration and metastasis. In addition, ACKR3 knockout using the crispr/cas9 method andS100A4knockdownusing targeted shRNA exerted additive effects suppressing cancer cell proliferation and migration. This exploratory analysis provides information about potential candidate genes. It also provides new insight into the molecular mechanism underlying docetaxel-resistance in androgen-independent prostate cancer and highlights potential targets to improve therapeutic outcomes.

## INTRODUCTION

Prostate cancer (PCa) is the leading nondermatological cancer and the second most common cause of cancer death among men in the western countries [1]. The primary treatment for metastatic PCa is androgen deprivation therapy (ADT). However, the tumors in most PCa patients become refractory to androgen deprivation treatment, and ultimately progress to androgen-independent prostate cancer (AIPC) [2].

Docetaxel (Doc), which was approved by the US FDA In 2014, remains the first-line chemotherapeutic agent for eligible patients with symptomatic AIPC [3]. AIPC patients taking Doc often achieve clinical remission with prolongation of life [4–6]. As a chemotherapy drug, Doc stabilizes microtubule structure by binding β-tubulin, thereby inhibiting DNA, RNA and protein synthesis, and thus impairing cell division [7, 8]. However, the efficacy of Doc is limited by chemoresistance. About 50% of patients respond poorly to Doc therapy, and some who initially respond well eventually exhibit Doc resistance [9–11]. It is therefore of major scientific and clinical interest to understand the mechanism underlying development of Doc resistance and to identify novel therapeutic targets for the treatment of AIPC.

Microarray technology has provided a wealth of functional information to investigate and identify novel targets for diagnosis of tumor progression [12, 13], while expression profile analysis can reveal primary genes associated with PCa development and resistance to chemotherapy [14]. In the present study, we used these tools with the Doc-resistant transcriptome microarray to identify genes differentially expressed between Doc-resistant prostate cancer cell lines and Doc-sensitive controls. The differentially expressed genes (DEGs) identified were then used for functional and pathway enrichment analyses. In addition, we used shRNA-mediated selective knockdown of S100A4 to investigate the synergistic effects of S100A4 silencing combined with ACKR3 knockout on PCa cell proliferation and migration. Our aim with all of these studies was to find better therapeutic strategies that overcome Doc resistance and enhance sensitivity to this chemotherapeutic drug.

## RESULTS

### Docetaxel sensitivity testing and DEGs in Doc-resistant PCa

**Figure 1:**
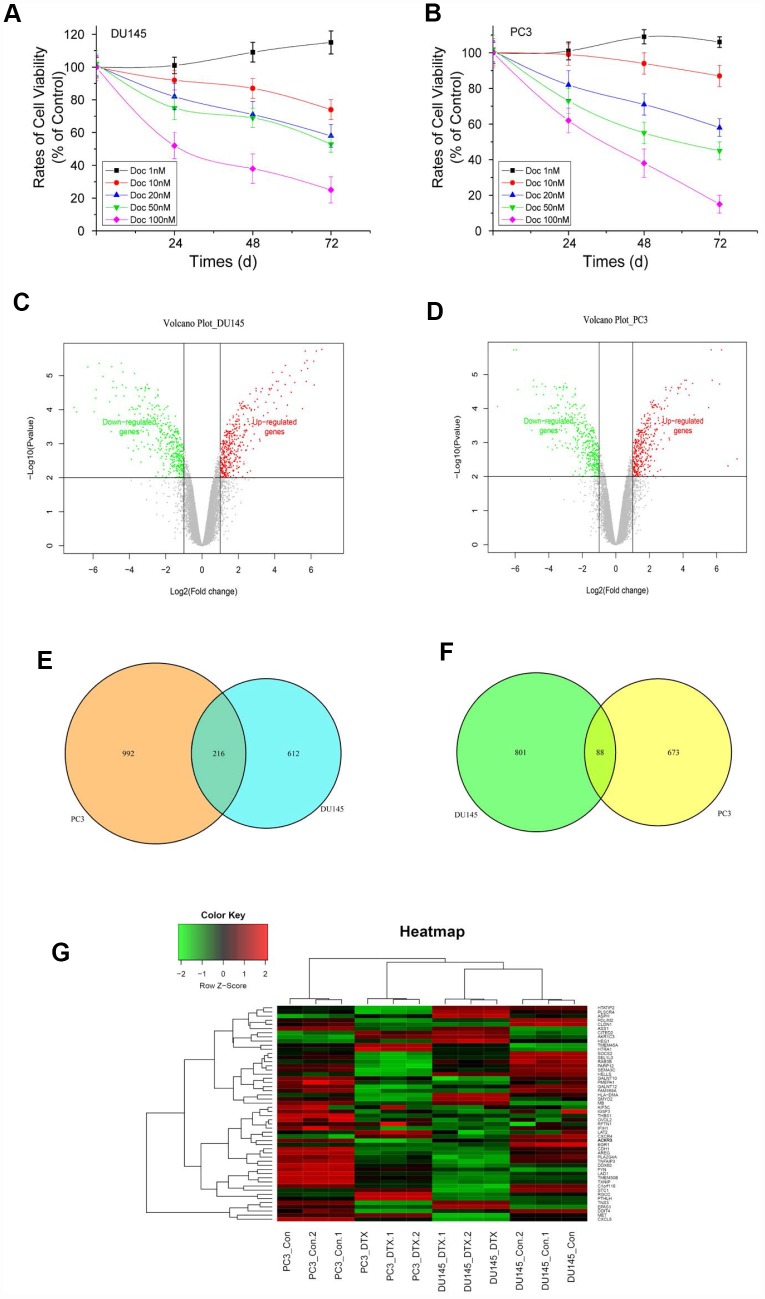
**Cell viability of PCa cells treated with different concentrations of Doc, volcano plots and Venn diagrams of DEGs.** (**A**) DU145 cells treated with different concentrations of Doc; (**B**) PC3 cells treated with different concentrations of Doc. Viability of DU145 and PC3 cells was determined by MTT assay. Error bars = SEM (n = 6). (**C** and **D**) Volcano plots of DEGs from DU145R and PC3R compared with their parent cell lines respectively. X-axes show the fold changes (log-scaled), and Y-axes indicate p values (log-scaled). Red and green dots represent upregulated and downregulated genes, respectively. Grey dots represent non-DEGs. (**E** and **F**) VennPlots for the downregulated and upregulated DEGs. (**G**) Heatmap of DEGs overlapping between the DU145R and PC3R datasets. Red represents higher expression and green lower expression. The criteria used to select DEGs were P<0.05 and |log2 (fold-change)|>1. DEGs, differentially expressed genes.

Cultured in increasing concentrations of Doc, at different time point, IC50 of DU145 and PC3 cells was determined by MTT assay. Results demonstrated that Doc decreased cell proliferation in a time and dose-dependent manner. The highest cytotoxicity of Doc was at 72h, and IC50 is 20nmol/L for the PCa cells (Figure 1A and 1B). To identify genes differentially expressed between Doc-resistant PCa cells (DU145R and PC3R) and their Doc-sensitive controls, threshold |logFC| >1 and P<0.05 were used as criteria for comparison. A total of 1719 DEGs were identified in DU145R cells, while 1970 DEGs were identified in PC3R cells (Figure 1C and 1D). TPMID: he top DEGs in DU145R and PC3R were presented in Supplementary Tables 1 and 2. Among those, 830 (DU145R) or 1208 (PC3R) were downregulated, and 889 (DU145R) or 762(PC3R) were upregulated. Comparison of the DEGs between two Doc-resistant cell lines revealed they shared 216 downregulated and 88 upregulated genes (Figure 1E and 1F). Thereafter, the overlapping DEGs were clustered to differentiate the Doc-resistant cells from their parental Doc-sensitive cells. From the overlapping DEGs, we found that S100A4 and ACKR3were dramatically upregulated while CDH1 was downregulated in both Doc-resistant cell lines. The heatmap of the overlapping DEGs is shown in Figure1G.

### Functional annotation of overlapping DEGs

**Figure 2:**
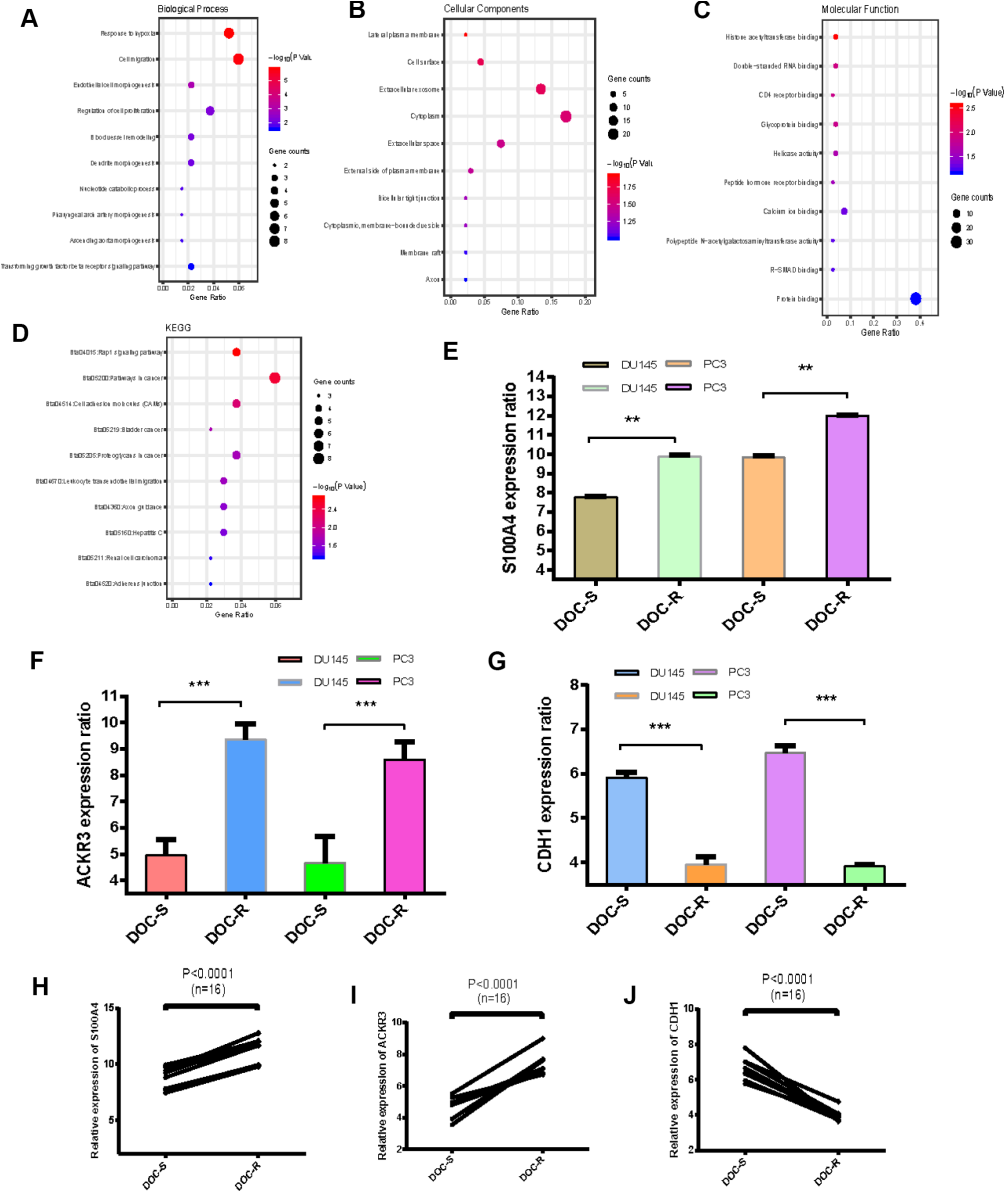
**GO and KEGG analysis of overlapping DEGs, expression levels of three critical genes.** (**A**–**C**) GO analyses. Shown are the top 10 biological processes (**A**), cellular components (**B**), and molecular functions (**C**). (D) KEGG pathway analysis. (**E**–**G**) Expression levels of S100A4, ACKR3 and CDH1 in Doc-resistant PCa cells (DOC-R) and Doc-sensitive controls (DOC-S). (**H**–**J**) Comparison of gene expression levels between Doc-resistant and Doc-sensitive cells. *P < 0.05, **P <0.01, ***P < 0.001.

The top five GO biological process terms associated with the overlapping upregulated and downregulated DEGs were involved in the response to hypoxia, cell migration, endothelial cell morphogenesis, regulation of cell proliferation and blood vessel remodeling (Figure 2A). In terms of cellular components, the DEGS were mostly associated with the lateral plasma membrane, cell surface and extracellular exosome (Figure 2B). Molecular function analysis indicated that the overlapping DEGs were mainly enriched in histone acetyltransferase binding, double-stranded RNA binding and CD4 receptor binding (Figure 2C). Subsequent KEGG pathway enrichment analysis revealed that the overlapping DEGs were primarily enriched in the rap1 signaling pathway and pathways involving cancer and cell adhesion molecules (Figure 2D). The top 5 enriched terms are presented in Table 1. These results suggest that the overlapping upregulated genes are mainly enriched in the regulation of cell proliferation and the TGF-signaling pathway, and that expression of S100A4 and ACKR3 is dramatically increased (Figure 2E to 2I). The expression patterns of S100A4 were also evaluated by qRT-PCR in Doc-sensitive (n=6) vs Doc-resistant (n=6) tumor samples from clinical PCa patients [Supplementary Figure 1A] as well as the cells of DU145, DU145R, PC3 and PC3R [Supplementary Figure 1B and 1C]. Results are consistent with the microarray data. The overlapping downregulated genes were mainly associated with regulation of cell migration. CDH1 expression, in particular, was markedly decreased (Figure 2G and 2J).

**Table 1:** GO functional and KEGG pathway enrichment analysis of DEGs.

**ID**	**Terms**	**Count**	**PValue**	**Genes**
**Biological process**				
GO:0001666	response to hypoxia	7	1.49E-06	EGR1, CXCR4, CD24, CITED2, DDIT4, TGFB2, MB
GO:0016477	cell migration	8	1.74E-06	HES1, TNS3, STYK1, FYN, CD24, THBS1, SDC2, TGFB2
GO:0001886	endothelial cell morphogenesis	3	1.05E-03	MET, HEG1, STC1
GO:0042127	regulation of cell proliferation	5	5.76E-03	PLA2G4A, STYK1, FYN, CXCL8, TGFB2
GO:0001974	blood vessel remodeling	3	6.65E-03	EPAS1, SEMA3C, TGFB2
**Cellular components**				
GO:0016328	lateral plasma membrane	3	2.23E-02	CLDN7, CLDN1, CDH1
GO:0009986	cell surface	6	4.46E-02	SLC1A3, CXCR4, IGSF3, MET, AREG, SDC2
GO:0070062	extracellular exosome	18	1.34E-01	LAD1, ASS1, PDLIM2, LGALS8, CDH1, CLDN11, LAT2, TFRC, SMPDL3B, PLSCR4, …
GO:0005737	cytoplasm	23	1.71E-01	EGR1, TXNIP, IFIH1, HTATIP2, EPAS1, SOCS2, ASS1, KIF5C, UPP1, PDLIM2, …
GO:0005615	extracellular space	10	7.44E-02	PTHLH, SMPDL3B, TGFBR3, CXCL8, SEMA3C, STC1, AREG, THBS1, QSOX1, TGFB2
**Molecular functions**				
GO:0035035	histone acetyltransferase binding	3	2.74E-03	EGR1, EPAS1, CITED2
GO:0003725	double-stranded RNA binding	3	1.25E-02	IFIH1, DDX60, RFTN1
GO:0042609	CD4 receptor binding	2	1.38E-02	PLSCR4, FYN
GO:0001948	glycoprotein binding	3	1.41E-02	FYN, CDH1, THBS1
GO:0004386	helicase activity	3	2.34E-02	IFIH1, DDX60, HELLS
**KEGG pathyways**				
bta04015	Rap1 signaling pathway	5	3.72E-03	MET, LPAR3, CDH1, THBS1, DOCK4
bta05200	Pathways in cancer	8	3.27E-03	EPAS1, CXCR4, MET, CXCL8, LPAR3, PTCH1, CDH1, TGFB2
bta04514	Cell adhesion molecules (CAMs)	5	7.17E-03	CLDN7, CLDN1, CDH1, CLDN11, SDC2
bta05219	Bladder cancer	3	1.71E-02	CXCL8, CDH1, THBS1
bta05205	Proteoglycans in cancer	5	1.83E-02	MET, PTCH1, THBS1, SDC2, TGFB2

### Modular analysis and pathway identification with PPI networks

**Figure 3:**
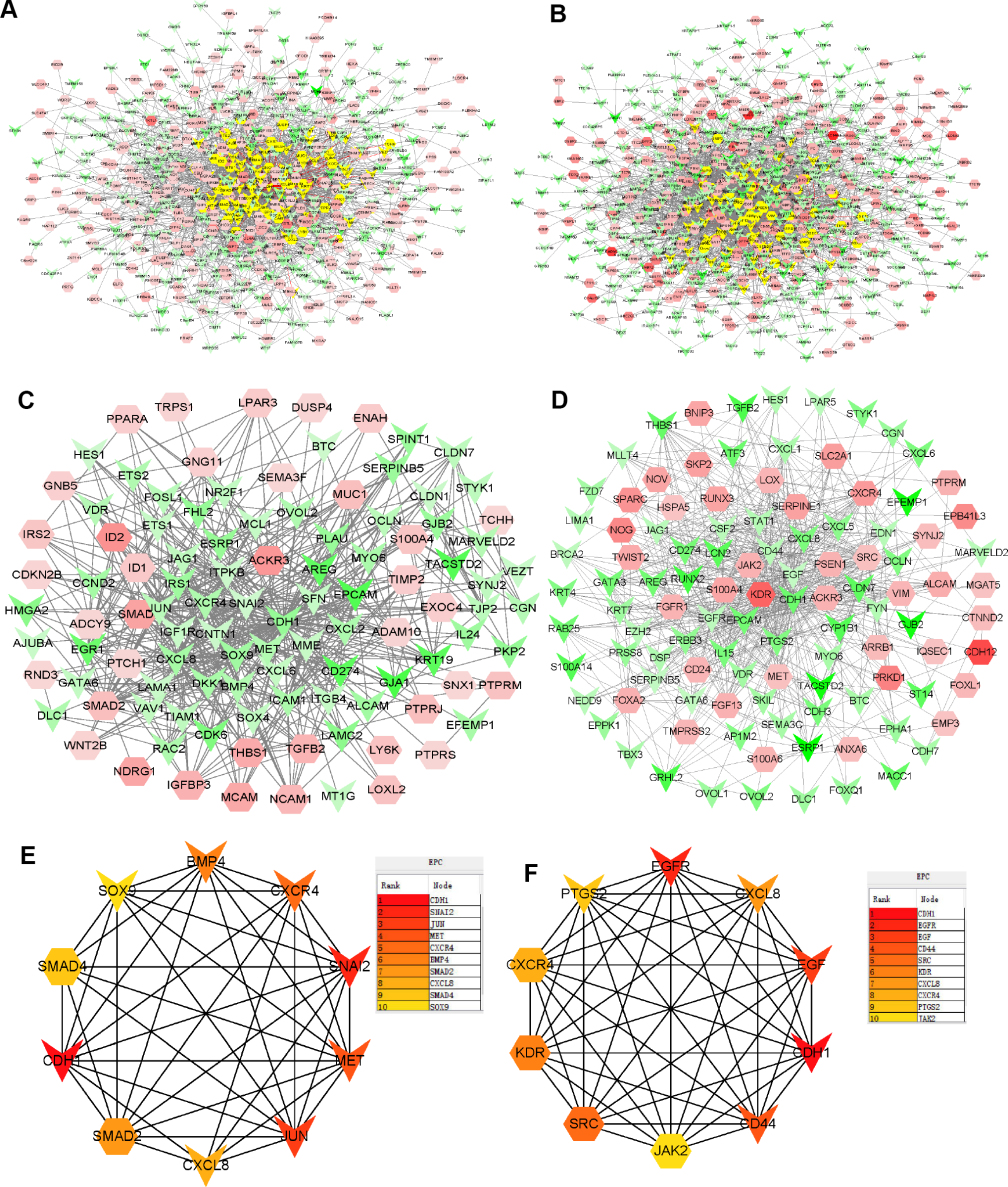
**PPI networks constructed using the DEGs from microarray data.** (**A**) Network of significant proteins from DU145R cells. (**B**) Network of significant proteins from PC3R cells. (**C**) Network derived from panel A with first-stage nodes associated with the core proteins S100A4, ACKR3 and CDH1. (**D**) Network derived from panel B with first neighbors associated with the core proteins S100A4, ACKR3 and CDH1. (**E**) Significant hub proteins extracted from network C. (**F**) Significant hub proteins extracted from network D. Red and green intensities indicate the degree of upregulation and downregulation, respectively.

To further identify the core genes contributing to Doc resistance, protein-protein interaction (PPI) networks were generated using significant DU145R and PC3R proteins (Figure 3A and 3B). To characterize the properties of the hub nodes based on analysis of the PPI network, we initially selected first-stage nodes associated with the core proteins S100A4, ACKR3, and CDH1 to identify candidate Doc-resistant PCa markers (Figure 3C and 3D). These consisted of 106 nodes and 556 edges in the DU145R network, and109 nodes and 672edges in the PC3R network. Functional annotation and pathway analysis of the nodes in the two networks are displayed in Table 2. After performing EPC (Edge Percolated Component) and shortest path analyses, the most significant modules composed of 10 nodes were screened out from the PPI networks, and the hub genes in the networks with a connectivity degree >16 were identified (Figure 3E and 3F). Comparison of the hub genes between the two Doc-resistant cell lines revealed CXCL8, CXCR4 and CDH1 to be common to both.

**Table 2:** The pathways enriched around the hub node in the PPI networks.

**ID**	**Terms**	**Count**	**PValue**	**Genes**
**DU145R**				
hsa04514	Cell adhesion molecules (CAMs)	9	6.52E-04	ALCAM, NCAM1, ICAM1, OCLN, CADM1, CD274, CDH1, ITGB2, SDC2
hsa04670	Leukocyte transendothelial migration	8	1.63E-03	ICAM1, OCLN, RAC2, CXCR4, MAPK13, ITGB2, PIK3R3, VAV1
hsa05200	Pathways in cancer	13	2.77E-03	BMP4, PLD1, EPAS1, STK36, MET, CDH1, SMAD2, TGFB2, IGF1R, RAC2, JUN, FAS, PIK3R3
hsa04650	Natural killer cell mediated cytotoxicity	8	3.23E-03	ICAM1, RAC2, ITGB2, FAS, PIK3R3, NFATC2, VAV1, SYK
hsa04664	Fc epsilon RI signaling pathway	6	5.58E-03	PLA2G4A, RAC2, MAPK13, PIK3R3, VAV1, SYK
hsa04062	Chemokine signaling pathway	9	5.89E-03	RAC2, ADCY9, CXCR4, CXCL2, GNB5, GNG11, CXCL6, PIK3R3, VAV1
hsa04370	VEGF signaling pathway	5	2.41E-02	PLA2G4A, RAC2, MAPK13, PIK3R3, NFATC2
hsa04510	Focal adhesion	8	2.78E-02	IGF1R, RAC2, JUN, MET, ITGB3, PIK3R3, THBS1, VAV1
hsa04350	TGF-beta signaling pathway	5	3.88E-02	BMP4, PPP2CA, SMAD2, THBS1, TGFB2
hsa04010	MAPK signaling pathway	9	4.17E-02	PLA2G4A, RAC2, MAPK13, JUN, MRAS, FAS, NFATC2, CACNA2D2, TGFB2
**PC3R**				
hsa04012	ErbB signaling pathway	10	9.66E-06	EGFR, CBLC, EREG, ERBB3, BTC, CAMK2B, AREG, EGF, NRG2, SRC
hsa04060	Cytokine-cytokine receptor interaction	14	2.76E-04	EGFR, CXCL1, CXCL5, IL6R, IL15, CXCL6, IL7R, TGFB2, KDR, VEGFB, IL23A, CXCR4, FAS, EGF
hsa05200	Pathways in cancer	14	7.37E-04	EGFR, FGF18, FGFR1, PTGS2, SKP2, BRCA2, FGF13, CDH1, BIRC3, STAT1, TGFB2, VEGFB, FAS, EGF
hsa04144	Endocytosis	11	7.37E-04	EGFR, CBLC, EPN3, CXCR4, ERBB3, EGF, IQSEC1, SRC, SH3GL2, F2R, KDR
hsa04630	Jak-STAT signaling pathway	9	3.53E-03	CBLC, SPRY2, IL23A, SOCS2, JAK2, IL6R, IL15, IL7R, STAT1
hsa04514	Cell adhesion molecules (CAMs)	7	2.02E-02	OCLN, CD274, CDH1, VCAN, ITGB2, CDH3, SDC2
hsa04520	Adherens junction	5	3.63E-02	EGFR, FGFR1, FYN, CDH1, SRC
hsa04510	Focal adhesion	8	4.47E-02	EGFR, VEGFB, FYN, EGF, BIRC3, THBS1, SRC, KDR
hsa04810	Regulation of actin cytoskeleton	8	6.03E-02	EGFR, FGFR1, FGF18, CHRM3, FGF13, ITGB2, EGF, F2R
hsa04010	MAPK signaling pathway	9	6.83E-02	EGFR, DUSP5, FGFR1, FGF18, FGF13, FAS, EGF, NFATC2, TGFB2

### Validation of overlapping DEGs

**Figure 4:**
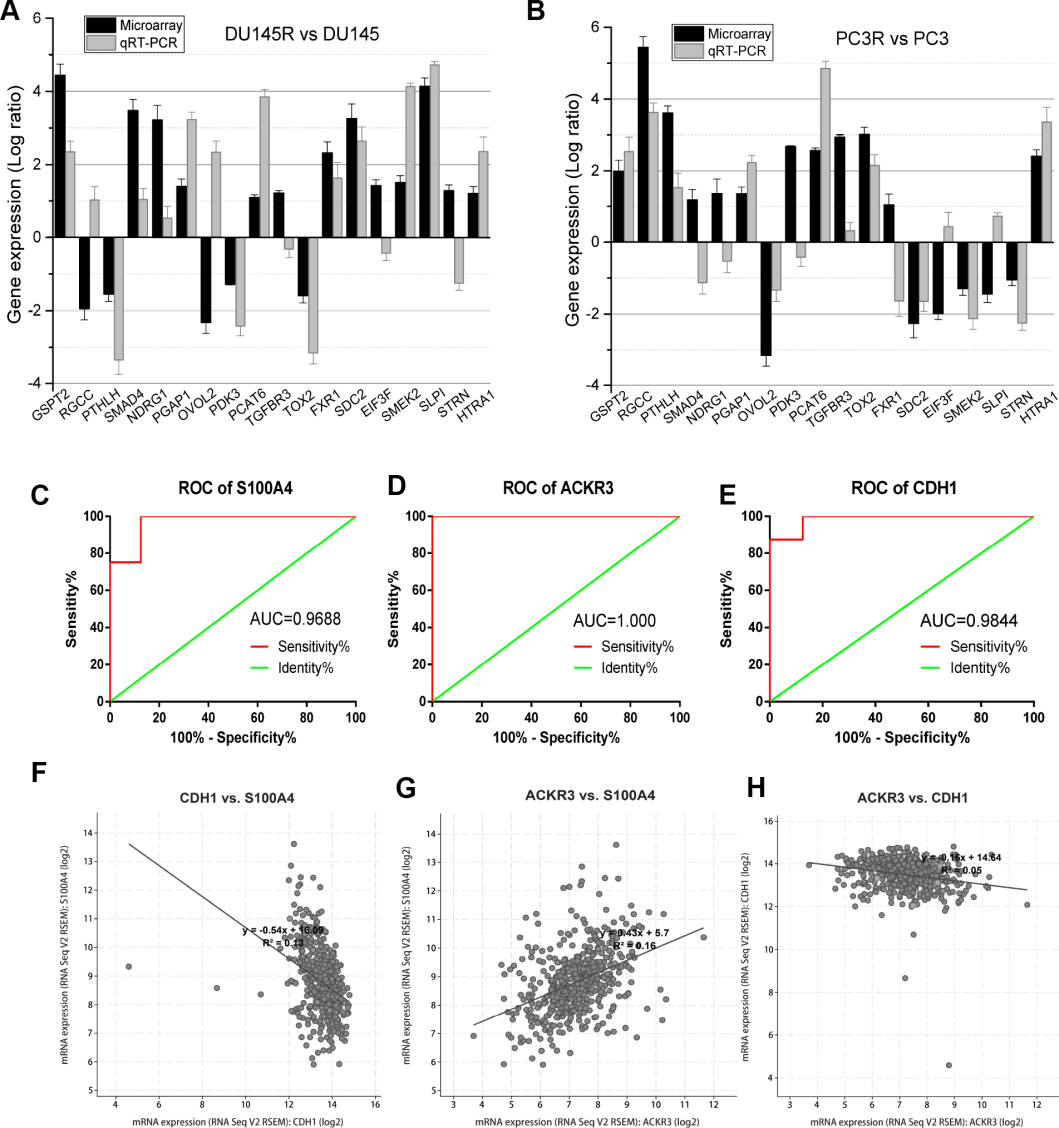
**Validation of DEGs identified in the microarray analysis.** (**A** and **B**) qRT-PCR analysis of 18 DEGs in Doc-resistant DU145R and PC3R smples. (**C**–**E**) ROC curves for S100A4, ACKR3 and CDH1 in the microarray. (**F**–**H**) Correlation between the expression levels among S100A4, ACKR3 and CDH1. Expression data are represented by a log ratio calculated by comparing ΔCq from the DOC-R samples with ΔCq from the controls. ΔCq was calculated as the difference between Cq of the targeted genes and Cq of the endogenous control gene ACTB.

To validate the microarray data, a panel of 18 DEGs randomly selected from DU145R and PC3R cells were compared to their respective controls using qRT-PCR (Figure 4A and 4B). The results showed that most of these genes had transcriptional profiles similar to those revealed in the microarray data. The Pearson correction coefficient between the microarray and qRT-PCR data for the 18 DEGs was 0.87. Thus, the microarray provided a reliable comparison of gene expression between Doc-resistant and Doc-sensitive prostate cancer. Receiver operating characteristic (ROC) analysis was then performed for S100A4, ACKR3, and CDH1. The areas under the ROC curves (AUC) for these three genes all indicated expression values of the microarray analysis for Doc-resistant PCa cell lines. The AUC for S100A4, ACKR3, and CDH1 were 1.000, 0.9688 and 0.9844, respectively (P<0.01, Figure 4C to 4E). Linear correlations among these genes are shown in Figure 4F to 4H. We also used a validation dataset containing 599 patients from TCGA to verify the correlation between the three genes. It showed that there is an inverse correlation between S100A4 and CDH1 expression, but a positive correlation between S100A4 and ACKR3, which is consistent with the analysis of the GSE33455 datasets.

### Western blot and IHC analyses

**Figure 5:**
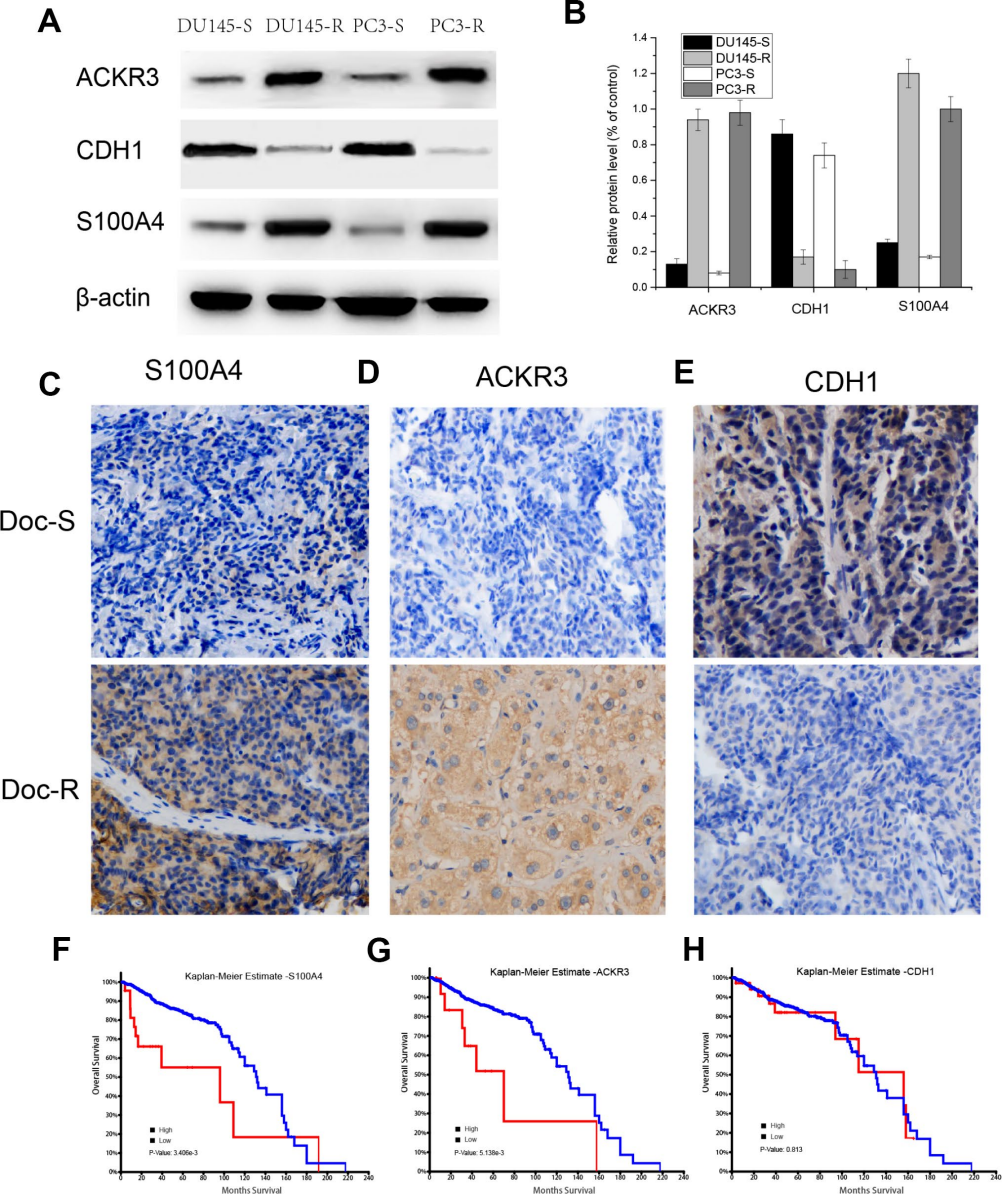
**Detection of S100A4, ACKR3 and CDH1 protein expression in Doc-R PCa samples, Kaplan-Meier curves of OS in patients from TCGA-PRAD.** (**A**) Western blots for S100A4, ACKR3 and CDH1 in DU145R and PC3R cells. (**C**–**E**) Immunostaining for S100A4, ACKR3 and CDH1 in representative samples of Doc-R tumor tissue from PCa patients. (**F**–**H**) Kaplan-Meier analysis of OS among patients in TCGA-PRAD dataset exhibiting high or low S100A4, ACKR3, or CDH1 expression.

Expression of S100A4, ACKR3, and CDH1 proteins in tumor samples from Doc-resistant and Doc-sensitive PCa patients was detected by western blotting and IHC. Upregulated expression of S100A4 and ACKR3 was detected in Doc-resistant tumor samples (Figure 5A and 5B). The elevated S100A4 and ACKR3 levels were detected predominantly in the cytosol and/or at the plasma membranes of cells in Doc-resistant tumors, while only weak expression of the two proteins was detected in Doc-sensitive tumors (Figure 5C and 5D). On the other hand, CDH1 was markedly downregulated in Doc-resistant tumor samples as compared to Doc-sensitive controls (Figure 5E). In addition, the effect of S100A4, ACKR3, and CDH1 expression on overall survival (OS) was assessed using the TCGA-prostate adenocarcinoma (PRAD) dataset (Figure 5F to 5H). Kaplan-Meier curves compared using log-rank tests showed that patients highly expressing S100A4 or. ACKR3 had significantly shorter OS, whereas the level of CDH1 expression had no significant impact on OS.

### ACKR3 knockout combined with S100A4 silencing synergistically inhibits Doc-resistant PCa cell viability and migration

**Figure 6:**
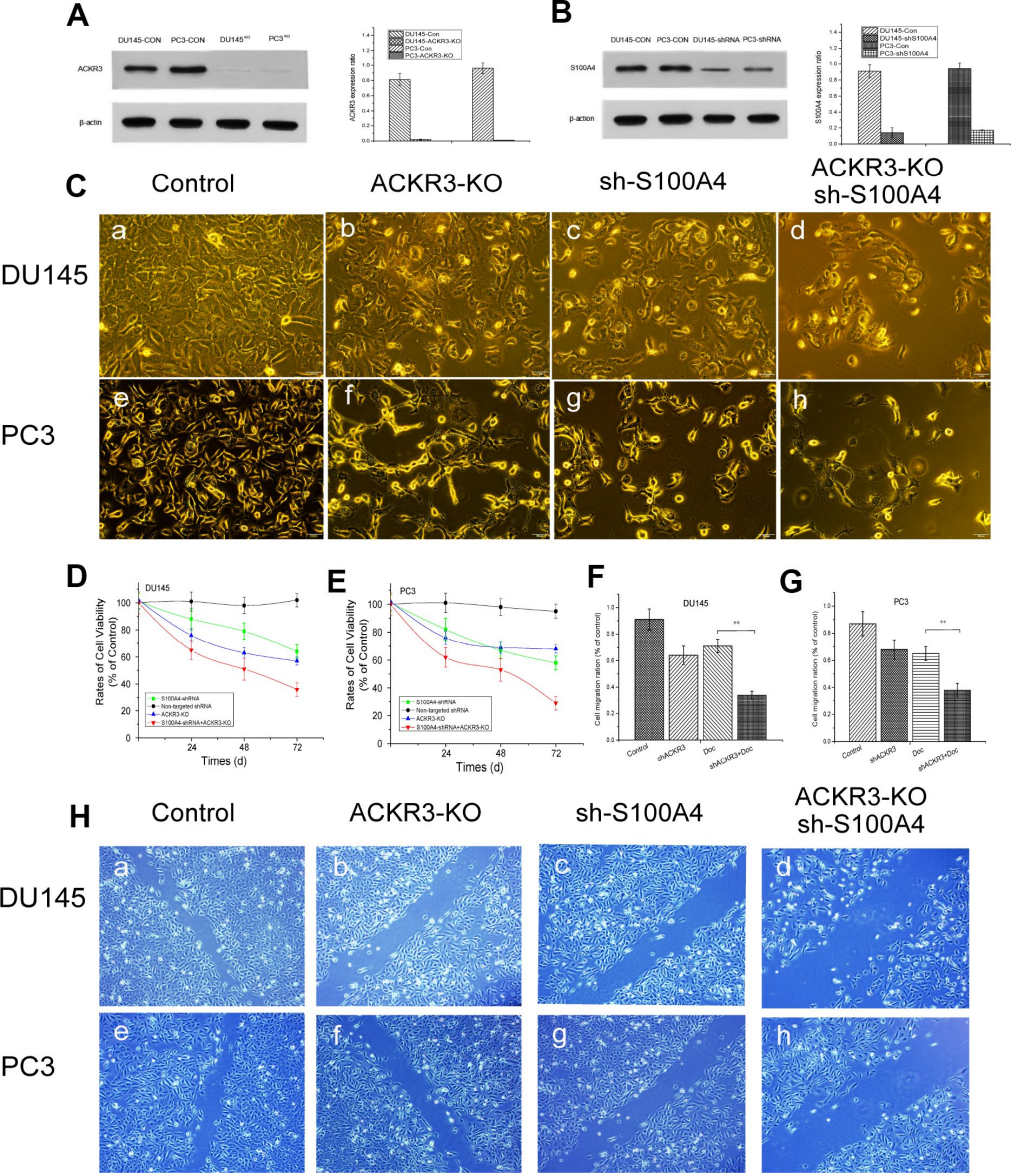
**Cell viability and migration effects after treatment with ACKR3 knockout and/or S100A4 knockdown.** Western blots (**A**) ACKR3 knockout and (**B**) S100A4 knockdown. (**C**) Photomicrographs of DU145R and PC3R cells 48 h after ACKR3 knockout and/or S100A4 knockdown. (**D** and **E**) Relative viability rates among DU145R and PC3R cells after 24, 48 and 72 h under the indicated treatment condition. Viability among control cells was assigned a value of 100%. (**F**) Photomicrographs of migration of Doc-resistant DU145R and PC3R cells in wound healing assays 48 h after ACKR3 knockout and/or S100A4 knockdown. (**G** and **H**) Relative quantification of migration in wound healing assays under the indicated treatment condition. Migration of control cells was assigned a value of 100%. *P < 0.05, **P <0.01, ***P < 0.001.

To better understand the effect of upregulated S100A4 and ACKR3 expression on Doc-resistant PCa cell viability and migration, ACKR3 was knocked out using the crispr/cas9 method in DU145R and PC3R cells and/or S100A4 expression of knocked down through transfection of targeted shRNA. After confirming the diminished expression of ACKR3 and S100A4 by western blotting (Figure 6A and 6B), MTT and SYTOX Green analysis were used to evaluate cell proliferation Wound healing assay was performed to assess cell migration. The results showed that ACKR3 knockout or S100A4 knockdown was sufficient to reduce Doc-resistant PCa cell viability (p<0.05, Figure 6C to 6E, Supplementary Figures 2 and 3). Moreover, combining the two treatments elicited a synergistic inhibitory effect, reducing DU145R and PC3R cell viability to 38% and 31% of control, respectively (p<0.01). The combined treatments also significantly decreased cell migration as compared to control (p<0.01, Figure 6F and 6H). These observations are consistent with the idea that ACKR3 and S100A4 overexpression contribute to the progression of AIPC.

## DISCUSSION

PCa is a heterogeneous disease that is a major health concern for men worldwide. A variety of approaches have been taken to elucidate the mechanism(s) underlying its development and to identify new therapeutic and prognostic targets [15–17]. Although chemotherapy is an important treatment for AIPC, its benefits are often limited by its side effects and the emergence of drug resistance. Genome-wide analysis has proven to be successful in a variety of experimental settings, and has the potential to reveal the dynamic molecular behavior ongoing during tumor progression [18]. Results of the gene expression profile analysis of the microarray data reported here reveal the DEGs shared by two Doc-resistant PCa cell lines and establish their association with various genetic networks and signaling pathways. ACKR3, S100A4, CDH1, CXCR4, and CXCL8 were identified as DEGs in both DU145R and PC3R cells. Moreover, they act as hubs in various PPI networks, indicating their potentially significant roles. CDH1 and CXCL8 were found to be down-regulated in both DU145R and PC3R cells. On the other hand, levels of CXCR4 expression were upregulated in PC3R cells, but downregulated in DU145R cells. CDH1 (E-cadherin) is a calcium-dependent adhesion molecule that plays key roles in cell growth and differentiation, morphological changes, and apoptosis in normal cells and tissues [19–21]. E-cadherin promotes epithelial cell adhesion, formation of stable intercellular connections, and maintenance of tissue structure and function [22]. Downregulation of E-cadherin expression can reduce the adhesive force between tumor cells, enabling cells to detach from the primary tumor, and increasing their invasive and metastatic potential [23, 24]. Nevertheless, we did not find a significant correlation between E-cadherin expression and OS in PCa patients.

We found that levels of ACKR3 and S100A4 expression were markedly increased in patients with AIPC. Moreover, Kaplan-Meier analysis showed their increased expression to be associated with poor OS. ACKR3, also known to as CXCR7, is an atypical chemokine receptor that belongs to the G protein-coupled receptor (GPCR) superfamily. Considerable evidence indicates that GPCRs do not operate as isolated proteins, but interact with other proteins that influence their trafficking and signal transduction properties within the plasma membrane [25–27]. ACKR3 is upregulated in a variety of pathological conditions related to infection, inflammation, and ischemia [28]. Altered expression of ACKR3 has also been detected in liver, prostate, kidney, and breast cancers [29, 30]. Several in vivo and in vitro studies have found that high levels of ACKR3 expression promote cell proliferation, invasive migration, tumor growth, and metastasis [31–33]. Higher expression of ACKR3 is also associated with poorer outcomes in terms of disease-free survival, and there is a positive correlation between high ACKR3 levels and cancer cell metastasis [34]. Several studies describe an association among the ACKR3-CXCR4 axis, disease progression and poor OS among cancer patients [35–38].

S100A4 belongs to the S100 family of EF hand calcium-binding proteins and has been shown to promote metastasis in a model system of breast cancer [39]. Given the heterogeneous properties of Doc-resistant PCa, investigation of the role of S100A4 in different PCa cell lines remains of great interest. Our study demonstrated that levels of S100A4 expression are significantly higher in Doc-resistant DU145R and PC3R cells than in their Doc-sensitive controls, which was consistent with the previous report [40]. The increased expression of S100A4 correlated positively with PCa progression, which was consistent with observations in other tumor types [41], and with poorer OS among patients. Like ACKR3, therefore, S100A4 may also play a key role in Doc-resistant PCa cell growth, invasion, and metastasis.

To further investigate the influence of S100A4 and ACKR3 on Doc-resistant PCa cell function, we combined ACKR3 knockout with S100A4 knockdown in DU145R and PC3R cells. We found that suppression of ACKR3 and S100A4 have synergistic inhibitory effects on Doc-resistant PCa cell viability and migration. ACKR3 is reportedly activated via β-arrestins and directly promotes Akt and MAPK activity, ERK phosphorylation, and activation of the JAK2/STAT3 pathway [42]. There is controversy regarding the involvement of ACKR3 in chemotaxis, with some reporting that ACKR3 itself is sufficient to induce migration of different cell types [43, 44], while others report indicated that S100A4 correlates with cancer development and plays an important role in cancer pathogenesis and metastasis [45]. Thus, the functions of ACKR3 and/or S100A4 in PCa progression await further clarification.

In summary, our findings demonstrate for the first time that ACKR3 and S100A4 are over expressed in Doc-resistant PCa. These two mediators exert synergistic effects that contribute to PCa progression and are predictive of a poor prognosis in patients with Doc-resistant PCa. Because the molecular mechanisms underlying the effects of ACKR3 and S100A4 in Doc-resistant PCa remain largely unknown, further studies exploring their mechanisms of action are needed. Nevertheless, we believe that our study is a valuable addition to the current research into PCa and provides additional perspective on AIPC.

## MATERIALS AND METHODS

### Cell treatments

The DU145 and PC3 human PCa cell lines were purchased from the American Type Culture Collection (ATCC; Manassas, Virginia USA). Cells were cultured at 37°C under 5% CO_2_ in Dulbecco's modified Eagle’s medium supplemented with10% fetal calf serum and 1% penicillin streptomycin (Gibco). Doc was purchased from R&D Systems, Sigma-Aldrich, and Enzo Life Science. Confluent cells were treated with Doc (1, 10, 20, 50 or 100nmol/L) for 24 h, 48 h and 72 h, respectively. Cells viability was measured by MTT assay. To generate Doc-resistant cells, DU145 and PC3 cells (1×10^6^ respectively) were incubated with docetaxel (10 nmol/L) for 24 hours, then Doc was removed and cells were incubated in complete DMEM medium. Cells were treated again immediately after cell growth was recovered. The total cycles of treatment were 20 with the time period of 6 months [46, 47].

### Gene expression profile datasets and DEG identification

The resultant Doc-resistant DU145R and PC3R cells compared with their respective controls DU145 and PC3were used for affymetrix microarray. Total tumor RNA was extracted using Trizol reagent (Takara, Dalian, China) and concentrations were determined by a spectrophotometer (NanoDrop, Nyxor Biotech). All the processes were carried out according to the manufacturers’ instructions. Enrichment of total RNA from samples was carried out using the RNeasy Micro kit (Qiagen, Germantown, MD, USA), and samples’ quality and quantity were assessed on a spectrophotometer. Hybridization was performed in Affymetrix Human Genome U133Plus2.0 array. CEL files in different databases were converted to expression measures and normalized using the affypackage in R [48]. The DEGs were subsequently calculated using the Limma package, based on the Benjamini and Hochberg procedure [49]. Genes differentially expressed between Doc-resistant and Doc-sensitive samples were defined using threshold |logFC| >1 and P<0.05 was used as criteria for comparison. Venn diagrams were generated using the VennDiagram R package.

### GO and KEGG pathway analysis of DEGs

DAVID (Database for Annotation, Visualization and Integrated Discovery http://david.abcc.ncifcrf.gov/) [50, 51] was used for Gene Ontology (GO) enrichment analysis. The DEGs in the Doc-resistant DU145 and PC3 human PCa cell lines were screened for functional enrichment. GO analysis was used to predict the degree of enrichment and the potential functions of the DEGs in biological processes, cellular components and molecular functions. In addition, Kyoto Encyclopedia of Genes and Genomes (KEGG) pathway enrichment analysis was used for systematic analysis of differences in gene functions. The false discovery rate, or q-value, was adjusted to 0.05, and P<0.05 was considered the cut-off criterion.

### Western blot analysis

Preparation of total cell lysates and the procedures for western blot analyses was essentially as described by Day et al [52]. Protein samples were separated on 10% polyacrylamide resolving gels and transferred onto nitrocellulose membranes for 2h at 250 mA. After incubating the membranes for 1h at 25°C in 5% (w/v) Marvel/PBS/3% (v/v) Tween-20 (PBST) to block nonspecific protein binding, the membranes were incubated overnight at 4°C with ACKR3, CDH1 and S100A4monoclonal antibodys (1:1000 dilution; Invitrogen, California, USA). The membranes were then washed three times for 10 min each in TBST and incubated for1h at 25°C with horseradish peroxidase-conjugated secondary antibody (Amersham Life Sciences, Buckinghamshire, UK). After another three 10-min washes in PBST, bands were detected using enhanced chemiluminescence (ECL+ reagents, Amersham). Densitometric quantification of band intensities was performed using Kodak one-dimensional image analysis software.

### Gene validation using qRT-PCR

A panel of 18 randomly selected DEGs compared with their respective controls was evaluated using quantitative real-time PCR performed by QPK-201 SYBR Green master mix (Toyobo, Osaka, Japan) and an ABI 7300 system from Applied Biosystems. The primers used were obtained from Invitrogen (Beijing China). The thermocycling protocol entailed an RT step at 50°C for 20 min, followed by a DNA polymerase activation step at 95°C for 2 min and 50 PCR cycles (95°C for 20 s, 60°C for 30 s). All reactions were conducted in triplicate. The fold-change in expression of each gene was calculated using the comparative C_T_ method. Expression data are represented by a log ratio calculated by comparing ΔCq from the Doc-resistant samples with ΔCq from the controls. ΔCq was calculated as the difference between Cq of the targeted genes and Cq of the endogenous control gene ACTB.

### Cell viability assay

The Doc-resistant DU145R and PC3R cells were transfected with S100A4-targeted shRNA (sh-S100A4) (Invitrogen, Shanghai, CN) and/or ACKR3 was stably knocked out using the crispr/cas9 method. Cell viability was assessed by incubating the cells in 100 μl/well MTT solution (0.5 mg/mL in PBS) for 3 h (at 37°C, protected from light), after which the supernatants were carefully removed and 150 μl of DMSO were added to each well. The cells were then shaken for 10 min in the dark. Absorbance was measured at 450 nm using a Microplate Reader (Bio-Rad 680). The cell proliferation and cytostatic rates were analyzed using GraphPad Prism 4. Untreated cells were used as controls.

### Immunocytochemistry

Immunohistochemistry (IHC) was carried out with 4-μm-thick sections of formalin-fixed, paraffin-embedded, Doc-resistant and Doc-sensitive tumor tissues from patients who had received transurethral prostatic resection before the start of Doc treatment. Following deparaffinization and rehydration of the tissue sections, antigen retrieval was performed by microwaving in 10 mM citrate buffer (pH 6.0). Primary anti-S100A4 antibody (Beijing Zhongshan Golden Bridge Biotechnology Co., Ltd., Beijing, China) was applied at 1:50 dilution. Peroxidase-conjugated, anti-rabbit secondary antibody was then applied at 1:500 dilution. The labeling was visualized using diaminobenzidine (DAB), and sections were counterstained with hematoxylin. After mounting, the sections were observed under an Olympus BX51 microscope at a magnification of 200×. At least four sections of tumor tissue were used for quantitative IHC.

### Wound healing assay

To assess the effect of ACKR3 knockout and/or S100A4 knockdown on PCa cell motility, DU145Rand PC3R cells were transfected for 4hwith S1004A-targeted shRNA or non-targeting control shRNA (Invitrogen, Shanghai, CN) and/or ACKR3 was knocked out using the crispr/cas9 method. For scratch assays, DU145R or PC3R cells (1×10^6^ cells per well) were seeded into 6-well plates and grown to 70% confluency, after which they were washed in sterile phosphate buffered saline (PBS) and ACKR3 was knocked out and/or S100A4 was knocked down as indicated above. The cells were then treated with 20 nmol/L Doc before the cell monolayer was physically wounded by scratching the surface with a pipette tip (1000 μl) as uniformly and straight as possible. Images of cells invading the scratch were then captured 0, 8, 16, 24 and 48 h later using a phase contrast microscope (IX 70 Olympus Optical Co., Germany). The pictures were evaluated by measuring the areas of the wounds using a Leica image analysis system (Leica, Mannheim, Germany). Migration rates, expressed as percent scratch closure, were calculated using the following formula: % scratch closure= a-b/a, where “a” is the initial distance between edges of the wound, and “b” is the distance remaining cell-free during cell migration to close the wound. The experiments were repeated at least two times in triplicate wells.

### Statistical analysis

All statistical analyses were carried out using SPSS 18.0 software. Data are shown as the mean± SD. Two-tailed Student’s t-tests were used to assess differences between means. Values of *P*<0.05 were considered significant.

## Supplementary Material

Supplementary Figures

Supplementary Table 1

Supplementary Table 2
