# Supplementary material for: Expression profile-based screening for critical genes reveals S100A4, ACKR3 and CDH1 in docetaxel-resistant prostate cancer cells
Source: Aging (Albany NY). 2019 Dec 29;11(24):12754–72. doi: 10.18632/aging.102600 (PMC6949054; doi:10.18632/aging.102600)
Supplement: Supplementary Figures [file aging-11-102600-s003..pdf]

SUPPLEMENTARY FIGURES

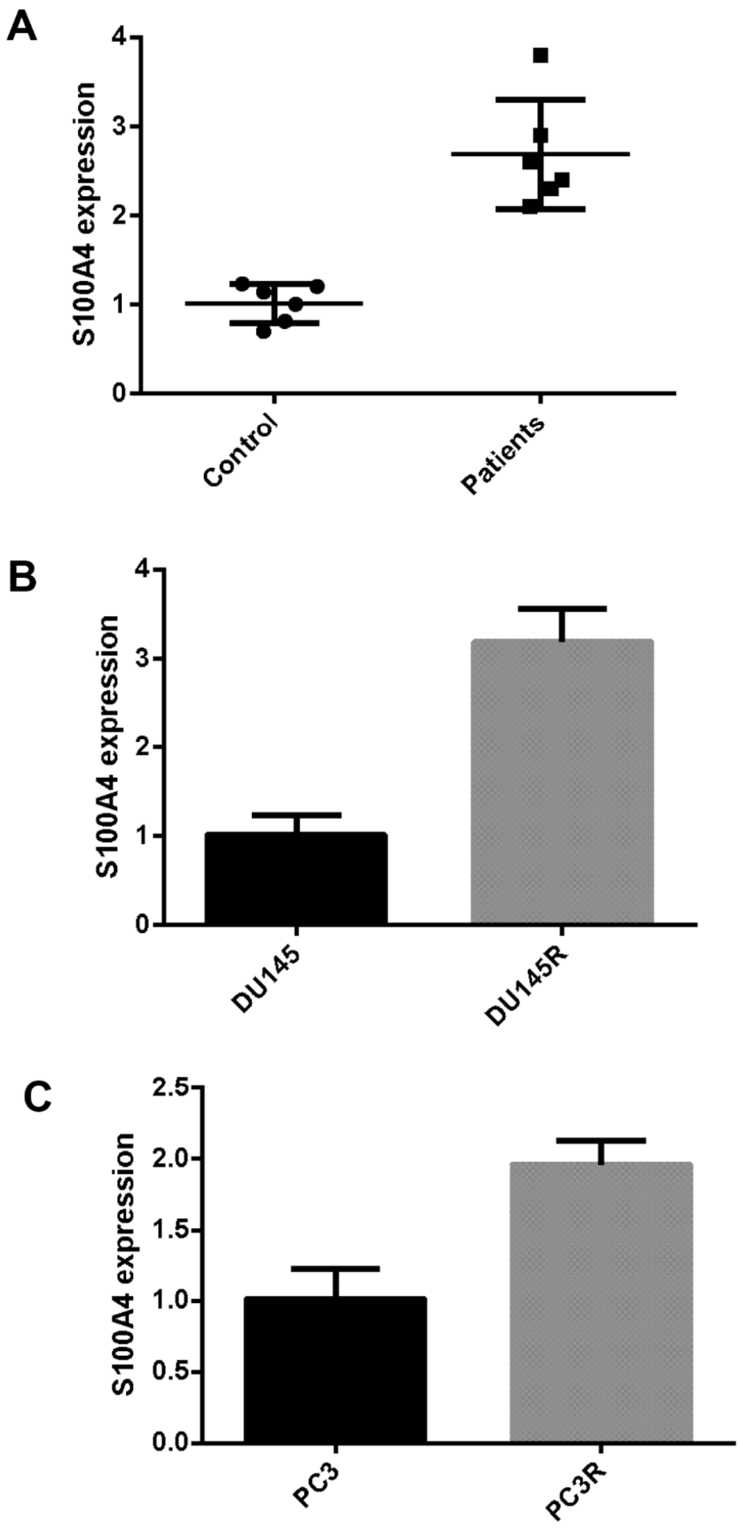

Supplementary Figure 1. Expression patterns of S100A4 were evaluated by qRT-PCR. (**A**) Samples from clinical PCa patients. (**B**) DU145 vs DU145R. (**C**) PC3 vs PC3R.

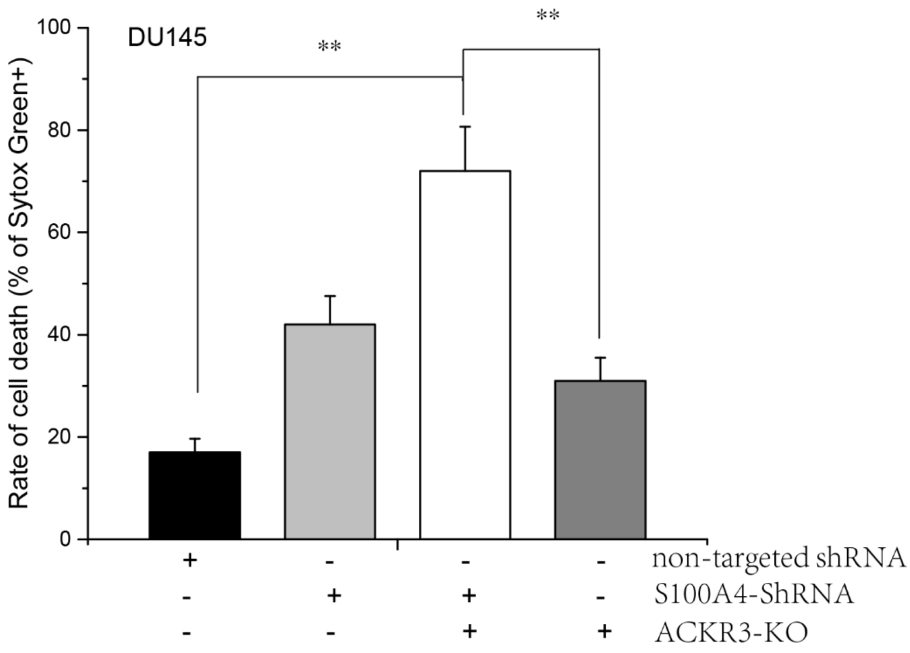

Supplementary Figure 2. Cell viability of DU145R after treatment with ACKR3 knockout and/or S100A4 knockdown. Cell viability was measured at 48 h of treatment and measured by sytox assay, 488 and 530 nm wavelengths were used for excitation and emission, respectively. *P < 0.05, **P <0.01, ***P < 0.001.

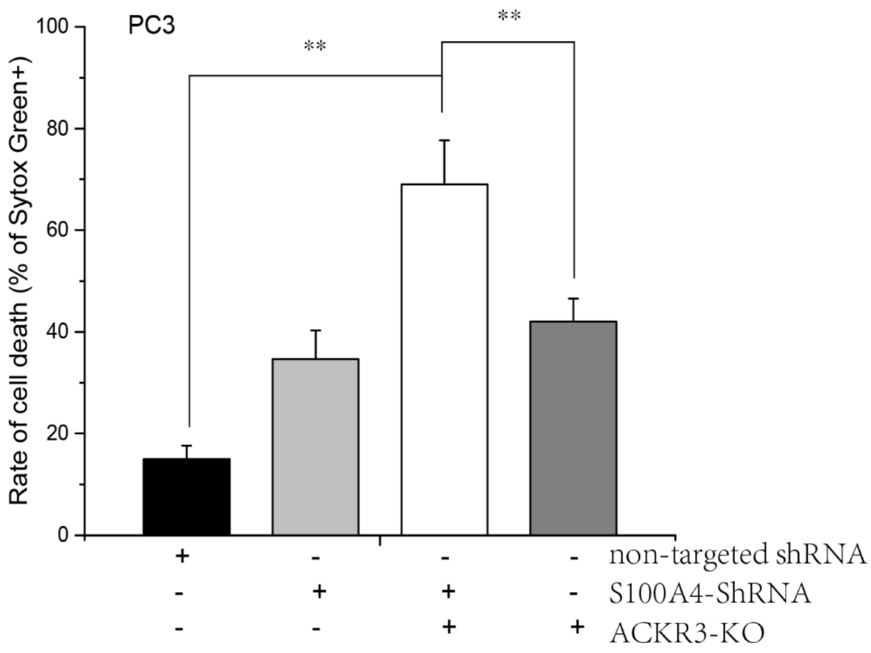

Supplementary Figure 3. Cell viability of PC3R after treatment with ACKR3 knockout and/or S100A4 knockdown. Cell viability was measured at 48 h of treatment and measured by sytox assay, 488 and 530 nm wavelengths were used for excitation and emission, respectively. *P < 0.05, **P <0.01, ***P < 0.001.
