# Supplementary material for: Expression profile-based screening for critical genes reveals S100A4, ACKR3 and CDH1 in docetaxel-resistant prostate cancer cells
Source: Aging (Albany NY). 2019 Dec 29;11(24):12754–72. doi: 10.18632/aging.102600 (PMC6949054; doi:10.18632/aging.102600)
Supplement: Supplementary Table 1 [file aging-11-102600-s001..docx]

**Supplementary Table 1. Top DEGs in DU145R.**

Top DEGs in DU145R

Gene logFC adj.P.Val

TKTL1 5.407870096 5.14E-13

EYA4 5.266872342 7.21E-13

NID2 5.077168059 1.45E-12

S100A4 4.984638182 3.72E-12

COL4A5 4.865020774 7.80E-12

CACNA2D1 4.771183956 1.80E-13

FBLN1 4.597937184 6.31E-11

ROBO1 4.540378252 3.42E-11

GSPT2 4.440879216 1.80E-13

ID2 4.177103866 8.28E-11

RSPO3 4.146277092 1.62E-10

SLPI 4.123876325 1.08E-10

TNNC1 4.048160708 4.59E-10

SMAD6 4.04021512 2.47E-12

AKR1C1 4.023285454 7.41E-13

ACKR3 3.963904291 6.97E-11

AKR1C2 /// LOC101930400 3.92443674 5.16E-13

GPR64 3.890272308 3.90E-12

ID3 3.736008202 1.19E-11

XAGE1B /// XAGE1E 3.698633674 1.04E-09

SMAD4 3.478734905 6.36E-10

MB 3.449092539 1.28E-10

AKR1C1 /// AKR1C2 /// LOC101930400 3.305965489 3.45E-11

SDC2 3.268359017 2.73E-11

FRMD3 3.227729668 3.61E-10

MCAM 3.213875072 3.02E-10

INPP4B 3.209666895 3.40E-11

ALPP 3.189175689 7.61E-10

PIK3C3 3.171936467 1.31E-11

THBS1 3.158144721 6.43E-10

OLFM1 3.118596949 7.97E-10

MMP28 3.098868037 3.83E-10

FOXL2 3.087818031 2.62E-11

EPAS1 3.04329769 2.63E-12

EPB41L5 3.03168016 3.37E-11

SDC2 3.027323505 1.30E-11

CASC10 2.934055994 2.86E-11

PLAG1 2.924808602 4.25E-10

FBLN1 2.923777523 7.44E-11

THBS1 2.907030449 1.72E-10

IGFBP3 2.902443263 3.27E-11

SYNE1 2.874575962 2.89E-10

RP11-140I16.3 2.842189592 8.27E-10

RPS20 /// SNORD54 2.818870496 6.82E-09

MCAM /// MIR6756 2.806341902 4.95E-08

ZIC2 2.795642621 5.61E-10

AKR1C1 2.794708686 1.51E-10

TGFB2 2.766822861 1.23E-10

CYP24A1 2.761731161 2.22E-10

HIST1H3A /// HIST1H3B /// HIST1H3C /// HIST1H3D /// HIST1H3E /// HIST1H3F /// HIST1H3G /// HIST1H3H /// HIST1H3I /// HIST1H3J 2.759942788 1.25E-10

AKR1C2 /// LOC101930400 2.75303825 4.18E-11

C8orf44 /// C8orf44-SGK3 /// SGK3 2.693237568 4.07E-10

TP53INP1 2.685894454 9.74E-11

TUB 2.645773353 2.71E-09

NR4A3 2.638805123 1.35E-09

NCAM1 2.632793352 9.66E-09

MMP28 2.605712727 2.89E-10

INPP4B 2.602877799 7.81E-11

LOC729887 2.598399594 8.22E-10

TGFB2 2.59688642 2.83E-08

LIMCH1 2.593537686 2.15E-08

JMJD6 2.587374391 1.34E-08

WFDC21P 2.583481679 4.58E-10

ZFP42 2.550829115 2.92E-10

CYP4F3 2.539283173 4.67E-10

ZNF704 2.537762759 2.32E-10

NCOA7 2.535041162 2.54E-10

TGFB2 2.527235109 6.53E-10

RP11-873E20.1 2.511698886 3.65E-09

IGFBP3 2.481705981 3.11E-08

LOC100506558 /// MATN2 2.481223365 7.35E-08

LIMCH1 2.474483172 2.92E-10

RBM24 2.469392834 2.09E-10

THBS1 2.462516035 2.81E-09

GOSR2 2.434413775 2.48E-08

CITED2 2.420655412 8.77E-10

MLLT11 2.401541108 4.88E-08

ADM 2.391830924 8.39E-10

ZDHHC11 /// ZDHHC11B 2.390588664 1.62E-10

TLE4 2.388002067 1.76E-08

CECR2 2.373821062 8.63E-08

CBLB 2.367822024 2.08E-08

SMAD4 2.367244818 1.11E-09

CEBPD 2.366069384 1.15E-08

HTATIP2 2.362859282 1.11E-10

TKTL1 2.357738946 7.19E-10

MUC1 2.356936088 5.93E-10

NR4A3 2.351293365 1.07E-09

ANKRD1 2.32864801 2.25E-07

FXR1 2.325895658 6.53E-10

SMAD2 2.321332421 2.54E-10

MIR612 /// NEAT1 2.316134995 4.41E-07

FADS3 2.316007684 1.41E-09

KLHL24 2.313702531 3.00E-09

PTPRJ 2.304717206 1.24E-09

SNHG19 2.300106777 2.14E-11

MCAM /// MIR6756 2.299133118 5.84E-09

TMEM165 2.293977738 9.08E-10

FRMD3 2.284545542 4.14E-09

TAGLN 2.284184752 1.12E-09

ATF7IP2 2.282999079 1.14E-09

EYA4 2.280421319 4.88E-06

MBNL2 /// MBNL2 2.273925685 2.23E-07

KRBOX1 2.267340906 2.36E-09

RP11-391M1.4 2.265199241 3.35E-11

CITED2 2.262427607 3.42E-07

C16orf45 2.254881603 2.08E-10

CBLB 2.251459803 2.54E-10

C10orf54 2.251274403 9.10E-09

SLCO4A1 2.244728529 3.76E-10

LOC153546 2.243594994 3.56E-06

ZNF704 2.236616347 1.14E-09

TYMSOS 2.236450924 9.44E-08

TLE4 2.236021009 3.87E-10

DAB2 /// LOC101926921 2.234729471 1.56E-10

FADS3 2.219355397 6.42E-11

RP11-48B3.4 2.21895824 1.83E-08

ANTXR1 2.211491808 4.15E-11

LOXL2 2.200756133 9.40E-10

IER5L 2.195688234 1.63E-10

ID2 2.194340597 1.13E-07

PTPRM 2.18724365 6.58E-10

PCDHB14 2.187055326 1.32E-09

GNAL 2.183351948 7.26E-08

RP5-1092A3.4 2.178322162 3.65E-08

LIMCH1 2.177951051 1.32E-08

SKIDA1 2.159709444 1.58E-09

KLHL24 2.154235666 1.11E-06

SCARA3 2.153545878 1.63E-07

SYNE1 2.147871524 8.58E-10

LY6K 2.145233058 1.66E-09

C9orf3 2.14270312 3.82E-08

RORA 2.136133428 2.36E-08

TMEM74 2.135316646 7.81E-10

RP4-758J24.5 2.125577141 9.09E-09

RP11-410L14.2 2.124122601 6.73E-10

LOC285147 2.111706524 8.73E-09

PLSCR4 2.109290455 6.30E-09

AMPH 2.087111076 5.09E-07

RPS15A 2.070601883 4.98E-10

PLD1 2.057700829 3.11E-09

POLI 2.053737081 9.50E-09

DNAJC15 2.052099933 1.55E-08

NANOS1 2.047315341 2.35E-08

KIF13A 2.044588581 2.96E-07

GNAL 2.042840517 1.70E-07

DLX1 2.040242538 1.10E-09

ABAT 2.034221162 1.52E-08

CCNG2 2.028269031 1.88E-07

RP11-554J4.1 2.027000737 5.69E-09

MIR612 /// NEAT1 2.01373344 2.09E-09

MIR612 /// NEAT1 2.011544834 2.85E-06

IRS2 2.007839011 1.98E-10

CHCHD7 2.005072117 2.07E-08

IRS2 2.002432228 7.82E-09

CCNG2 2.002429322 6.53E-10

TENM3 2.002418072 7.27E-07

TMEM45B -2.000168568 6.25E-07

AK021804 -2.002670679 3.73E-08

CDK17 -2.007955722 1.04E-09

DMKN -2.008515989 1.07E-10

CGN -2.011920027 3.81E-10

CDK17 -2.013423919 9.09E-10

ACOX2 -2.018947919 1.47E-08

AREG -2.020471933 5.89E-07

JUN -2.021420965 8.86E-10

NFATC2 -2.021864815 1.50E-09

TSPAN7 -2.025116367 3.50E-07

MCTP1 -2.026449777 1.49E-07

ARNTL2 -2.027088361 2.36E-09

COMMD3 -2.027443807 2.02E-10

IL11 -2.030089215 1.91E-08

FHOD3 -2.037276169 6.43E-10

CLEC2B -2.038165649 1.09E-06

FOSL1 -2.041917037 4.62E-08

DQ592230 /// RP4-555D20.2 -2.045958345 1.02E-07

SLAIN1 -2.04797632 7.96E-09

LOC541472 -2.049410721 1.39E-08

MREG -2.057126429 4.55E-10

LOC400043 -2.058810886 9.33E-08

BTBD11 -2.062772558 1.26E-07

TNFAIP3 -2.063973037 9.75E-09

ZBTB20 -2.067510493 1.14E-06

ACO1 -2.070529292 2.22E-10

MIR100HG -2.072565354 3.67E-09

MICAL2 -2.077709884 1.52E-09

FRMD4B -2.077877856 7.74E-08

RBM47 -2.07990949 3.24E-08

AX747517 /// CST6 -2.085491348 1.14E-09

CASP4 -2.08804673 3.90E-10

BLACAT1 -2.092476192 1.74E-07

CALD1 -2.093413955 2.22E-10

EREG -2.093979346 1.32E-08

DENND2D -2.099668291 1.52E-10

ELOVL2 -2.106947218 8.57E-09

ARHGEF26 -2.108518872 2.11E-08

BNC1 -2.113393102 4.62E-08

RARRES3 -2.113879724 1.14E-07

PADI2 -2.11496917 9.88E-10

LRCH2 -2.11944805 1.32E-09

CALD1 -2.11945183 2.71E-09

KLRC1 /// KLRC2 -2.122086225 1.05E-09

CGN -2.134011383 1.57E-08

LOC100996579 -2.136631006 2.01E-06

KCNQ3 -2.137006236 3.44E-07

VDR -2.138123588 2.92E-07

MICAL2 -2.155547973 6.97E-10

PLEKHA5 -2.157486524 3.02E-10

GBP1 -2.15874525 8.92E-10

TRIM38 -2.164142836 4.51E-08

MARVELD2 -2.164919732 7.96E-09

VWDE -2.167441679 4.58E-09

ALCAM -2.175169059 1.23E-08

EXPH5 -2.175956138 3.86E-08

COL5A2 -2.177349586 4.84E-09

PPARD -2.177796103 8.91E-09

SCNN1A -2.17836229 6.45E-09

LOXL1-AS1 -2.187785277 2.70E-09

PTPLAD2 -2.197153238 3.05E-07

ERAP2 -2.20212088 1.08E-07

ITGA2 -2.202610033 5.56E-10

ZBTB20 -2.203070665 6.15E-07

ESRP1 -2.203984607 1.42E-08

SYK -2.206112273 1.69E-08

FRK -2.207625175 3.02E-09

LAMA3 -2.208524533 2.41E-10

CHRNB1 -2.208672607 2.93E-07

CPED1 -2.213665694 2.37E-08

CPVL -2.224169873 8.13E-10

STC1 -2.239430822 1.16E-06

RTEL1-TNFRSF6B /// TNFRSF6B -2.250703478 1.52E-09

CARD6 -2.258790334 3.61E-10

MYO10 -2.261639121 9.92E-08

CD274 -2.263272361 2.98E-08

DKK3 -2.264777319 3.81E-10

BMP4 -2.27034309 4.16E-09

MET -2.273034909 3.38E-11

TAOK3 -2.274934272 8.05E-10

COCH -2.282078481 8.13E-09

SMAGP -2.284811433 3.87E-10

STEAP1 -2.290830306 5.22E-11

HOXB6 -2.291135271 1.14E-09

SH3RF2 -2.292128706 1.04E-10

PLA2G4A -2.297430226 8.05E-08

ETS2 -2.299450002 1.49E-09

IL31RA -2.31049013 1.23E-08

MRPL52 -2.313400811 7.97E-10

OVOL2 -2.325863645 1.87E-09

ZBTB20 -2.333743613 1.67E-07

DLC1 -2.33995268 1.45E-07

SYK -2.347376584 2.96E-07

SPOCD1 -2.349599306 7.97E-10

MAPK13 -2.350477545 3.07E-11

ICAM1 -2.353594632 8.28E-11

STRIP2 -2.365043663 1.12E-08

EXPH5 -2.365355964 1.50E-09

TNFAIP3 -2.370759332 2.04E-09

ARHGEF28 -2.372140206 4.92E-10

COL27A1 -2.379038403 3.94E-08

AK021804 -2.379842688 4.35E-10

ADAM19 -2.390910032 4.92E-09

RAC2 -2.391597645 6.36E-10

CXCR4 -2.397476865 5.16E-10

NT5E -2.400582736 1.46E-11

ARL4C -2.407042657 4.39E-09

SLAMF7 -2.408990656 6.54E-09

FRMD4A -2.416507047 3.26E-06

IL31RA -2.423356658 6.55E-09

TNS4 -2.425625642 4.98E-10

CXCL2 -2.428662706 3.00E-10

KRTAP2-3 /// KRTAP2-4 -2.439917405 6.43E-10

ICAM1 -2.457442113 1.28E-09

EFEMP1 -2.465983317 1.23E-10

DPYSL3 -2.486311774 2.92E-08

MYO6 -2.500536589 4.51E-10

MPZL2 -2.530577693 1.58E-08

STRIP2 -2.543490979 3.57E-08

CXCL8 -2.546042779 7.21E-11

IRS1 -2.550939619 1.13E-08

UPP1 -2.554763545 1.17E-09

MYO1D -2.555239033 7.82E-08

DKK1 -2.56363879 8.66E-12

ARHGEF26 -2.58532947 2.00E-09

BC005081 /// RP11-284F21.10 -2.591475009 4.89E-09

EFEMP1 -2.597221411 1.20E-08

COCH -2.599443101 3.20E-08

IFIH1 -2.61581745 8.15E-09

SDR16C5 -2.620830908 6.18E-09

LOXL1 -2.624738357 2.23E-09

NPAS2 -2.632164119 1.31E-06

CXCL8 -2.634747391 1.30E-08

GATM -2.638894589 1.40E-07

MX1 -2.63969588 3.91E-09

GBP1 -2.655798314 7.10E-09

SOX9 -2.668375756 6.86E-11

F2RL1 -2.695407061 6.49E-09

CCND2 -2.696989803 2.92E-10

TMCC3 -2.708141195 2.77E-09

CDK6 -2.725751031 6.43E-10

GATM -2.729882809 1.23E-08

LTB -2.753939577 2.10E-09

ALCAM -2.766145633 1.60E-11

ALCAM -2.780882903 2.33E-12

PLAU -2.788128479 9.12E-12

GBP3 -2.799492403 9.28E-11

ERAP2 -2.800270845 3.93E-11

LINC00973 -2.803328612 3.93E-11

ITGBL1 -2.808792074 1.11E-10

NPAS2 -2.812474836 8.85E-11

ITGBL1 -2.816436639 1.82E-08

MARCKS -2.824264348 1.55E-11

SOX9 -2.828082286 8.74E-10

ANK3 -2.838731609 3.06E-09

PEAR1 -2.840073074 3.40E-11

L3MBTL4 -2.849539041 6.36E-10

QSOX1 -2.853303224 2.09E-10

ITGB2 -2.859478516 6.64E-11

LGALS3BP -2.863456989 4.11E-09

IL24 -2.869321609 3.00E-09

ADAMTS6 -2.873038973 2.00E-09

IRS1 -2.873122394 1.30E-10

ERAP2 -2.875632531 1.02E-09

LAMC2 -2.883380094 1.72E-09

CD302 /// LY75 /// LY75-CD302 -2.897485862 2.26E-11

FRMD4A -2.907164472 2.99E-08

MAP7 -2.912722124 8.33E-08

DKK3 -2.917909776 1.02E-10

PSMB9 -2.927585324 9.85E-12

PBX1 -2.951068803 1.22E-09

HAS3 -2.958141795 8.28E-11

GALNT6 -2.960798789 3.98E-09

SERPINB5 -2.962591097 3.93E-11

TRIM2 -2.963970099 3.51E-09

COL13A1 -2.987659796 6.31E-11

PADI2 -2.990916126 2.75E-10

GJB2 -2.998456203 3.59E-10

SPINT1 -2.998784025 1.46E-07

SAMD9 -3.002410746 4.14E-08

BTBD11 -3.020349124 1.17E-08

KCNMA1 -3.035088836 3.02E-10

DUSP6 -3.077708873 3.93E-11

PLAU -3.091613924 1.14E-09

ZBED2 -3.122561398 3.38E-11

TNC -3.14394598 2.47E-12

ESRP1 -3.146787651 7.96E-10

TMEM156 -3.1637915 3.70E-11

FHL2 -3.168494637 6.50E-12

ITGB2-AS1 -3.185904403 1.80E-09

PKP2 -3.206666322 1.45E-11

GOLT1A -3.260671371 9.29E-08

LINC00511 /// LINC00673 -3.30229035 7.61E-11

EMP1 -3.304639632 3.38E-11

SLC1A3 -3.310455001 5.50E-09

CLDN11 -3.332956876 1.96E-10

CST6 -3.361391123 1.23E-10

LOC101060391 -3.400611968 1.49E-10

SYTL2 -3.403285546 3.94E-12

LAMC2 -3.450807652 1.66E-11

SYTL2 -3.495425471 6.35E-12

GALNT3 -3.495941435 1.71E-12

HMGA2 -3.533289055 9.16E-12

SPNS2 -3.540186449 4.65E-12

STK31 -3.549959115 2.63E-11

FGFBP1 -3.551064785 2.31E-10

TRIM2 -3.565252828 1.20E-12

GALNT6 -3.581034442 6.64E-11

G0S2 -3.616950257 3.22E-12

DUSP6 -3.625674456 3.22E-12

CDK6 -3.655958158 3.06E-11

LOC344887 -3.702806761 1.75E-10

TMCC3 -3.705609993 1.31E-10

RAB38 -3.757570831 3.65E-10

CDH1 -3.760176467 6.50E-12

GPR87 -3.770245865 5.59E-11

MTUS1 -3.839380594 7.14E-11

CDK6 -3.88202693 5.16E-10

JPH1 -3.975713056 4.12E-12

MYO10 -3.998352734 4.24E-13

BCL2A1 -4.015056892 7.20E-11

C6orf141 -4.046678725 2.07E-12

EGR1 -4.096463422 3.38E-11

PXDN -4.261373524 4.65E-12

DUSP6 -4.282247922 2.36E-13

STC1 -4.3380355 3.37E-12

SERPINA1 -4.412763788 1.42E-11

PPAPDC1A -4.500525069 9.64E-13

EGR1 -4.54543136 2.14E-11

VAMP8 -4.552725439 9.85E-12

MTUS1 -4.576933161 4.42E-13

LETM2 -4.593790202 4.24E-13

MYEOV -4.641746895 7.46E-13

F13A1 -4.738358065 2.15E-11

PXDN -4.766164388 5.61E-09

STC1 -4.775450892 1.09E-11

F2RL1 -4.778204556 9.29E-13

SCEL -4.780895822 5.84E-09

RNF128 -4.847441231 1.48E-12

AIM1 -4.875447687 2.31E-13

SCEL -4.885787647 1.40E-09

AREG -4.904440595 2.44E-13

CD274 -5.039384512 6.56E-11

KRT19 -5.090676624 1.22E-14

TACSTD2 -5.273782773 7.80E-12

SERPINA1 -5.324473498 1.80E-13

EPCAM -5.385778663 2.05E-12

GJA1 -5.694783355 4.28E-12

MAL2 -5.735900756 1.51E-12

HSPA1A /// HSPA1B -6.298004348 3.82E-13

SCEL -6.409927973 1.21E-12

BCHE -8.080316354 3.11E-15
