# Supplementary material for: Expression profile-based screening for critical genes reveals S100A4, ACKR3 and CDH1 in docetaxel-resistant prostate cancer cells
Source: Aging (Albany NY). 2019 Dec 29;11(24):12754–72. doi: 10.18632/aging.102600 (PMC6949054; doi:10.18632/aging.102600)
Supplement: Supplementary Table 2 [file aging-11-102600-s002..docx]

**Supplementary Table 2. Top DEGs in PC3R.**

Top DEGs in PC3R

Gene logFC adj.P.Val

CADM2 5.019003111 7.83E-13

CCDC141 4.952696022 9.27E-12

SLC14A1 4.893939624 9.24E-13

CDH12 4.678482947 1.35E-11

VSIG1 4.486614319 1.89E-12

TTN 4.310576795 4.46E-12

PTPN5 4.276634834 7.81E-13

CADM2 4.213594121 5.75E-13

FBN2 4.112475014 1.81E-12

KDR 4.003777354 1.48E-11

LYZ 3.953812491 3.83E-12

GBX2 3.830440787 9.45E-11

C14orf37 3.779877633 5.13E-10

NAP1L3 3.68195019 6.63E-11

DPP4 3.675160076 3.45E-11

TMEM200C 3.64743074 2.85E-10

PTHLH 3.608939559 5.68E-11

DPP4 3.604536268 4.82E-09

S100A4 3.597284777 8.18E-11

SLC5A3 3.580852438 1.52E-11

CST1 3.570053062 5.61E-11

MIR612 /// NEAT1 3.507242434 5.61E-09

ACKR3 3.496523974 6.42E-11

DPF3 3.442945858 7.39E-11

LOC101927870 3.394049293 6.62E-08

C15orf65 3.33180317 6.52E-11

RBP4 3.311586993 2.76E-10

DHRS9 3.283433404 2.15E-10

APCDD1L 3.278663596 3.30E-10

HEY1 3.276419619 3.82E-10

DPP4 3.268197521 9.64E-09

SIGLEC15 3.220373504 2.46E-10

DHRS9 3.190357057 2.69E-10

CHST2 3.175618224 1.18E-11

PRKD1 3.159203864 1.48E-10

DPF3 3.141365415 7.64E-10

RRAGD 3.122385188 1.82E-10

ABHD12B /// MIR4454 3.111467734 9.00E-10

BCL10 3.087699431 2.32E-09

SLC5A3 3.082556124 1.00E-10

MIR612 /// NEAT1 3.04847267 3.80E-08

NOS1 3.016727936 2.44E-11

TOX2 3.015764243 6.06E-09

UBE2QL1 2.962738016 5.27E-09

CADM2 2.961679707 1.54E-10

NOG 2.956513582 2.29E-11

TGFBR3 2.940514703 1.88E-11

SCARA5 2.937394707 5.28E-07

HEY1 2.916629918 8.07E-11

C5orf42 2.911934692 7.92E-09

EPB41L3 2.880192127 3.25E-10

TGFBR3 2.867251183 1.20E-09

UBE2QL1 2.840162951 4.95E-10

LINC00520 2.807663855 1.20E-10

SCARA5 2.789315695 2.83E-10

PTGER2 2.779330747 3.24E-07

TMPRSS3 2.759587391 5.32E-09

KIAA1462 2.756761406 1.17E-09

MIR612 /// NEAT1 2.751637133 8.55E-11

LOC100996668 /// ZEB1 2.72998029 2.88E-11

DPYSL4 2.726763392 2.32E-08

EPB41L3 2.71142213 3.97E-10

SPARC 2.69596599 2.49E-09

PDK3 2.682130703 1.69E-10

RGS4 2.673963093 4.56E-10

RIN2 2.650296297 1.51E-08

RGS17 2.636178685 1.38E-10

TNXA /// TNXB 2.631783594 2.46E-10

CADM2 2.628938836 1.70E-09

NPAS3 2.623642141 3.94E-09

RDH10 2.616196788 1.13E-09

TNXA /// TNXB 2.608043289 3.08E-10

OSBP2 2.60494107 2.11E-09

SERPINE2 2.579154655 2.14E-11

HSD17B2 2.569352341 1.85E-10

PCAT6 2.5680275 1.76E-09

FGFBP1 2.566156059 4.46E-09

B4GALT6 2.564941096 3.57E-09

PLSCR4 2.550084251 7.64E-10

PCDH9 2.526558154 2.72E-09

TNXA /// TNXB 2.523056891 1.34E-09

RBM25 2.519889642 9.82E-09

KIAA1211 2.519055045 3.57E-11

CXCR4 2.51832422 2.73E-10

LOC101927870 2.508394988 7.35E-10

LOC100996668 /// ZEB1 2.504525877 1.49E-09

KIAA1462 2.487580286 1.61E-08

ST3GAL6 2.48548684 2.16E-07

RDH10 2.485069859 1.50E-10

PDK3 2.484848222 6.35E-10

CTD-2124B8.2 2.482462012 1.01E-09

TMEM158 2.472893396 9.57E-11

FCGR2A 2.467920105 5.54E-09

CLU 2.467764001 9.72E-11

GEM 2.44991608 8.25E-11

KLHL15 2.442714763 5.16E-11

SLC2A1 2.438870971 1.91E-11

LPIN2 2.435575722 4.28E-10

IL1RL1 2.434173104 2.21E-10

CTD-2124B8.2 2.432721575 2.02E-09

LOXL4 2.428272937 2.04E-09

PLA1A 2.425728073 9.32E-08

ADAMTS1 2.412143673 5.53E-09

RIMKLA 2.408525929 8.07E-11

HTRA1 2.406756114 3.97E-09

NPEPL1 2.404364307 1.85E-10

EPB41L3 2.400833881 1.17E-09

B4GALT6 2.399845848 1.46E-08

PDK3 2.397689633 3.11E-09

RRAGD 2.388126463 5.97E-09

PDK3 2.386990386 2.08E-10

COX7B2 2.380096246 2.04E-09

CLU 2.370948802 1.25E-09

SKP2 2.352541406 4.09E-08

EGFEM1P 2.34745247 4.79E-08

EBF2 2.335894561 7.85E-09

CEACAM6 2.325945476 9.14E-09

SERPINE1 2.312266838 2.26E-07

CADM2 2.305984128 2.29E-09

TCP11L2 2.305307996 2.30E-07

NPAS3 2.301149486 9.29E-09

TSNARE1 2.300074553 8.68E-09

EOMES 2.286005828 5.23E-10

SSTR5-AS1 2.285639735 4.79E-05

DGKE 2.27874384 1.67E-09

KIAA1462 2.273324337 6.07E-09

CLU 2.269000768 6.11E-09

ST3GAL6 2.267117205 2.04E-10

SCUBE3 2.266058066 3.50E-08

SLC5A3 2.263479349 9.36E-10

RNF144A-AS1 2.251481998 1.19E-09

IL6R 2.250998158 8.79E-08

CST4 2.244879575 3.70E-08

CLEC2B 2.241822324 3.51E-07

CD55 2.24123471 2.89E-10

PITPNC1 2.237891271 2.18E-07

VSIG1 2.237449815 2.92E-07

RUNX3 2.237389907 1.84E-08

MGC4294 2.231073458 3.50E-10

KLK10 2.23090992 9.29E-09

ANKRD36B 2.227888549 2.23E-06

AP1S2 2.227685653 7.64E-11

SKP2 2.227322541 1.75E-07

DGKE 2.220945416 5.29E-07

NOV 2.219933845 1.14E-05

RBM25 2.215586112 6.28E-07

CD55 2.213766897 1.02E-09

MAN1A1 2.212984847 1.10E-07

LOC101060604 /// SLC7A5P1 /// SLC7A5P2 2.207656763 4.64E-09

KLHL24 2.204212447 7.34E-07

AP1S2 2.199771521 1.29E-10

LOC100505817 2.194860719 1.08E-07

TMEM165 2.19271256 1.20E-09

SMAD9 2.178605942 2.60E-09

EN1 2.177845136 1.13E-08

SEMA6D 2.175432487 6.35E-07

SLC5A3 2.169463985 1.33E-10

AHNAK2 2.167933645 1.86E-10

GHR 2.167888994 1.28E-09

TTC6 2.163326207 2.92E-07

CAMK2B 2.160995702 1.47E-06

RASSF8 2.156842471 5.26E-09

PRSS35 2.150581018 2.06E-08

MSI2 2.14038997 6.14E-08

TFEC 2.128806019 4.16E-09

GFPT2 2.117924075 7.30E-08

SKP2 2.117245664 7.43E-08

PPP2R2C 2.109983785 1.70E-07

PHEX 2.100012583 1.91E-07

TWIST2 2.08481382 2.40E-10

CEACAM6 2.081995037 8.99E-08

KCNK1 2.079837174 3.11E-10

GPR153 2.079720957 1.92E-07

STC1 2.079514026 3.38E-08

LMBRD2 2.075025209 3.08E-09

SESTD1 2.069037258 2.29E-08

SLC2A13 2.068455379 9.29E-09

GLUL 2.067351631 2.11E-09

LPIN2 2.057918656 2.73E-10

ANKRD36BP2 2.057574364 1.12E-08

KCNMA1 2.05700228 4.95E-09

DUSP5 2.056278565 2.79E-08

OSBP2 2.044455243 1.21E-06

RDH10 2.041733453 3.63E-10

GCAT 2.034061566 3.91E-10

FAM155A 2.031559303 4.63E-08

SLC2A1 2.007296408 1.47E-08

MS4A7 -2.006053475 7.64E-10

HOXA3 -2.00729221 6.87E-10

AX747630 -2.007820637 6.01E-07

TC2N -2.012110433 1.21E-06

MGP -2.013038429 2.31E-07

SKAP2 -2.02239922 5.32E-09

FAM198B -2.022892365 1.84E-09

CCDC85A -2.022896469 7.82E-07

VDR -2.025280745 8.14E-08

SKAP2 -2.028685188 1.12E-09

FGF18 -2.030931613 3.64E-07

ACSM3 -2.032226035 1.57E-07

SLC27A2 -2.033726088 7.72E-08

SCN9A -2.035242994 1.83E-08

COMT -2.036111483 2.19E-10

TBX3 -2.036775707 1.06E-06

CXCL1 -2.038180448 1.83E-09

NEDD4 -2.038497771 2.78E-08

C4orf33 -2.04575549 2.12E-07

FAM221A -2.047336982 1.16E-09

C1orf210 -2.047845115 3.10E-06

ITGB2 -2.048160669 1.44E-09

BTG3 -2.059192634 8.20E-10

LPXN -2.068806835 4.73E-09

PHF14 -2.074704402 7.89E-11

PRR15L -2.079531107 4.14E-09

GPR126 -2.082344224 1.21E-07

IGSF9 -2.083336893 9.76E-10

BTC -2.089508328 1.92E-07

TMC5 -2.09395832 1.49E-09

PTPN20B -2.096715753 1.14E-08

HDAC9 -2.099981872 2.36E-08

GAD1 -2.100677756 1.52E-08

MOCOS -2.102664788 2.41E-10

CDH7 -2.104920324 5.32E-09

MYO6 -2.107547519 1.96E-09

PLAC8 -2.111597562 1.71E-06

SNX10 -2.118088199 1.55E-08

FGD3 -2.120123205 2.39E-10

SEMA3C -2.123378573 5.12E-11

BID -2.123818166 7.75E-10

MIR424 /// MIR503HG -2.124021274 2.62E-08

GABRB3 -2.124369628 2.27E-08

LOC101927137 /// SCLT1 -2.130994846 2.46E-09

CDC42EP3 -2.131894536 5.54E-10

MPP7 -2.136446422 2.04E-09

FLRT3 -2.139554535 2.59E-08

EPS8L1 -2.141224653 7.05E-09

TNFAIP8 -2.142945599 4.89E-09

DDX58 -2.142998437 1.01E-07

CHEK1 -2.143258763 1.27E-09

CHEK1 -2.144085884 3.11E-10

GJA5 -2.152805431 5.98E-09

OASL -2.159913754 9.65E-08

ACOXL -2.16052742 3.87E-08

IFI44 -2.161007337 4.94E-08

ZSWIM7 -2.164074748 3.97E-10

TTC22 -2.167205312 1.16E-07

ANKRD22 -2.172185357 4.07E-09

C11orf1 -2.175997893 3.11E-10

FRMD4A -2.179103492 4.23E-07

MAPK13 -2.179637753 5.68E-11

ARHGEF26 -2.180840147 1.15E-08

GLIPR1 -2.182004283 2.72E-07

GUCY1B3 -2.193438826 6.61E-08

GPR64 -2.195800535 8.86E-10

MESP1 -2.205199146 2.47E-11

PTGFRN -2.206334009 3.94E-10

DNAJC12 -2.207717521 5.93E-09

FZD7 -2.209116495 8.88E-08

AMIGO2 -2.211255562 1.16E-08

WWC1 -2.214731662 3.17E-11

SDC2 -2.216952833 9.95E-10

SRPX2 -2.217365982 6.94E-09

SLC16A7 -2.227909119 2.52E-08

ARHGAP8 /// PRR5-ARHGAP8 -2.228042535 4.51E-09

LOC101927204 -2.229199188 4.05E-10

VDR -2.229759704 1.55E-07

RASEF -2.249297601 9.73E-09

IL7R -2.249694887 3.59E-09

SLITRK6 -2.253963504 6.83E-09

HNMT -2.254839886 7.66E-10

GXYLT2 -2.255759581 1.28E-07

MRPL15 -2.256473407 9.21E-11

SLITRK6 -2.256621709 4.47E-08

PMEPA1 -2.260098093 1.85E-10

SDC2 -2.265154068 1.96E-10

FAM84B -2.268331233 1.44E-10

FGF18 -2.271803866 4.66E-08

SKIL -2.278468129 4.10E-10

C5orf46 -2.281303216 3.92E-07

CAPS -2.28497626 3.53E-08

SLC44A2 -2.293380393 2.69E-09

HAPLN1 -2.296678342 2.98E-08

CCDC71L -2.304947823 1.45E-09

SAMD9 -2.305568464 3.03E-08

C6orf132 -2.310539655 6.62E-10

HOXC6 -2.314420917 6.50E-10

AQP3 -2.316419959 4.05E-09

MNS1 -2.321373655 1.15E-07

FAM111A -2.323265897 4.13E-09

LOC729680 -2.327520396 1.94E-10

IL1R2 -2.330616985 3.41E-08

PSPH -2.336421773 1.15E-10

KRT7 -2.33656151 3.99E-10

GAD1 -2.339324157 5.57E-09

DLC1 -2.340386401 6.25E-09

TMC4 -2.351004328 8.74E-08

SLC51A -2.352837076 1.13E-08

SLC6A11 -2.361873017 5.66E-09

BIRC3 -2.365682834 3.68E-09

TMEM45B -2.365865948 3.57E-09

IGFBP5 -2.371959164 8.14E-07

EPN3 -2.372631369 4.47E-08

GALNT12 -2.373974006 4.18E-10

CDS1 -2.385394423 9.63E-10

DAPP1 -2.389355287 9.02E-09

GPR126 -2.39350103 6.52E-11

TBC1D30 -2.394246505 1.95E-08

FOXQ1 -2.402324975 9.87E-09

CLDN11 -2.407969902 3.76E-09

EPHA1 -2.408804986 1.80E-09

CDS1 -2.411058876 1.60E-09

TRBC1 -2.412515167 2.98E-09

OVOL1 -2.413022971 5.82E-10

DERA -2.41591121 2.14E-11

C11orf70 -2.418631959 1.02E-08

MICAL2 -2.420242369 2.93E-10

MARVELD3 -2.422320582 2.18E-07

CDC42EP3 -2.423827484 5.37E-11

ELOVL6 -2.423975589 1.30E-09

EPS8L1 -2.430930956 2.66E-09

EHF -2.432590493 3.99E-10

INHBA -2.43333599 4.09E-07

IFIT2 -2.436019897 6.50E-09

AF198444 -2.436629871 4.28E-10

ZDHHC21 -2.440171822 1.01E-10

GRAMD1C -2.440529687 2.34E-09

DHRS2 -2.441776511 3.92E-08

LITAF -2.445350981 5.23E-10

WWC1 -2.446886188 1.56E-07

TNFSF18 -2.453470727 1.92E-06

ANKEF1 -2.454878764 8.26E-10

CLEC7A -2.455945386 2.87E-09

EPPK1 -2.459058953 3.39E-08

IRF6 -2.459893537 5.83E-10

EPPK1 -2.462292126 9.23E-10

C9orf152 -2.468917139 1.37E-07

CGN -2.470917711 4.65E-11

MICAL2 -2.478341071 1.56E-10

INHBA -2.47841653 2.71E-08

DNAJC12 -2.48382379 1.03E-08

NOX5 -2.487167391 3.54E-09

FXYD3 -2.494370291 6.02E-08

DDX60 -2.506552025 4.48E-08

VCAN -2.507132232 2.93E-09

VGLL1 -2.516940196 5.97E-09

ARHGAP28 -2.517613242 2.35E-08

IL33 -2.521933306 7.06E-09

COL12A1 -2.522214871 3.58E-10

PARP12 -2.523590299 5.82E-09

BTG3 -2.530334696 3.40E-10

HOOK1 -2.531593369 3.30E-10

ARHGAP18 -2.535848715 1.11E-09

AX747517 /// CST6 -2.536928757 1.51E-10

CXADR -2.536970265 2.34E-10

C9orf64 -2.548340546 8.26E-10

WDR72 -2.55144156 1.06E-09

LNX1 -2.552059174 6.47E-09

PLAC1 -2.558224049 6.34E-09

PRSS8 -2.558485345 1.10E-08

C1orf116 -2.57238196 4.45E-10

COL2A1 -2.577340464 1.50E-09

ST14 -2.57899041 6.34E-09

OGFRL1 -2.579959465 3.39E-09

ERBB3 -2.581138636 2.09E-09

VCAN -2.593626656 3.14E-11

ENTPD3 -2.600797743 1.93E-09

TNFAIP8 -2.60869552 1.58E-09

CAPS -2.613389326 2.37E-10

LINC01116 -2.621382587 9.92E-11

SLC27A2 -2.624068916 5.82E-09

MB -2.624374348 1.54E-09

TIGD2 -2.62561718 1.51E-08

TGM2 -2.645540957 1.90E-11

BTG3 -2.648609912 3.47E-09

CA13 /// LOC100507258 -2.654042773 5.25E-09

KLK6 -2.655086318 9.76E-10

SERPINB7 -2.658297189 4.23E-08

IFIT2 -2.66122008 1.23E-09

CLEC7A -2.661953105 2.01E-09

DAPP1 -2.666450967 5.90E-09

TGM2 -2.684067888 9.08E-09

LOC100506098 -2.686060903 8.06E-10

TRIM6 -2.693383205 2.29E-10

KRTAP1-5 -2.696309614 3.10E-07

ERBB3 -2.704091261 2.00E-11

IGF2 /// INS-IGF2 -2.713863308 3.62E-09

GUCY1A3 -2.717337058 1.92E-11

MAP2 -2.723227504 4.32E-10

CDC42EP3 -2.730277422 3.77E-11

TBC1D30 -2.732450357 1.82E-10

ZNF165 -2.747129968 6.42E-10

OCLN -2.754038492 8.92E-09

AP1M2 -2.760680175 5.68E-11

XK -2.763890701 4.19E-10

ARL14 -2.77007776 9.29E-09

IL20RA -2.779195391 1.07E-09

SH3GL2 -2.798531776 1.50E-09

DSE -2.801339117 5.26E-10

CXADR -2.810717468 6.62E-11

LAD1 -2.812164888 6.58E-11

IFI44 -2.818003914 3.63E-09

LOC101927204 -2.821365175 5.11E-09

IFI44L -2.828282992 4.76E-07

PTGFRN -2.840466945 5.93E-10

TGFB2 -2.842067712 1.59E-08

BSPRY -2.848125311 1.59E-08

CXCL8 -2.857696106 2.05E-11

CXCL5 -2.861820637 1.25E-09

IL1RN -2.862758528 2.34E-10

MACC1 -2.886690397 2.46E-10

ARHGAP8 /// PRR5-ARHGAP8 -2.891235056 1.35E-11

DPYD -2.893962563 1.89E-08

TOX -2.895031699 3.63E-10

SH2D3A -2.895834218 4.75E-09

TRIM16 -2.903123467 1.90E-11

NOX5 -2.911411498 4.18E-09

MAL -2.949679632 1.48E-10

CAMK2N1 -2.967707356 2.22E-08

ATP2C2 -2.973485765 4.88E-10

PRRG4 -2.973998762 2.96E-10

AKR1B10 -2.977058432 2.83E-10

INPP4B -3.002296909 1.81E-11

TGM2 -3.015247026 2.73E-10

STYK1 -3.015685977 3.36E-09

CXADR -3.019089967 1.44E-10

THBS1 -3.024653626 3.05E-10

CYP1B1 -3.05203144 3.30E-10

ILDR1 -3.053285791 4.93E-09

AP1M2 -3.061513042 2.46E-10

C11orf52 /// HSPB2 /// HSPB2-C11orf52 -3.061741964 1.35E-11

LITAF -3.06946032 2.05E-12

ZDHHC23 -3.069522511 2.46E-10

ELOVL7 -3.070357286 1.15E-10

COL12A1 -3.085254965 1.88E-11

ALPP -3.099597847 8.26E-10

RBMS3 -3.101219914 2.02E-09

KRT4 -3.114411646 2.14E-11

KMO -3.115016924 2.13E-08

PTGS2 -3.131155687 1.49E-10

OVOL2 -3.152898228 8.25E-11

IFIT1 -3.154248469 3.91E-10

THBS1 -3.165252139 8.61E-11

OCLN -3.168249412 2.59E-12

NAT1 -3.189971488 4.23E-10

TMEM30B -3.201984722 5.12E-11

MPZL2 -3.205528888 5.61E-11

IGFBP2 -3.210723254 1.54E-09

SLC44A3 -3.22066243 4.75E-11

ARRDC4 -3.222669612 1.29E-10

SAMD12 -3.223041041 4.79E-11

GUCY1A3 -3.227980863 5.33E-11

AREG -3.230452462 4.44E-09

TMEM45B -3.237252751 4.18E-09

BSPRY -3.238962737 3.24E-09

IRF6 -3.241892771 1.33E-09

KMO -3.256696943 1.14E-09

CXCL6 -3.257172246 4.33E-10

HAPLN1 -3.272274013 6.52E-11

MPZL2 -3.292042251 5.16E-11

CXCL8 -3.307251624 1.09E-09

EHF -3.323082356 6.44E-12

ANKRD22 -3.323505194 1.38E-10

PEG3 -3.339628548 1.00E-10

IL15 -3.343870311 2.34E-10

CCBE1 -3.344820396 1.14E-10

EPS8L1 -3.345081514 1.25E-11

CDH3 -3.35167261 6.30E-11

THBS1 -3.361582976 2.95E-10

HOXA10-AS /// MIR196B -3.365216836 1.89E-12

BICC1 -3.373289508 7.44E-10

CAMK2N1 -3.431304522 2.28E-09

ARAP2 /// LOC101928667 -3.453483016 1.85E-10

GATA3 -3.455892856 2.95E-09

VCAN -3.456045761 6.63E-11

CD274 -3.466692558 2.05E-09

INPP4B -3.489010638 1.47E-11

TSPAN2 -3.553749153 1.53E-11

EPCAM -3.557993423 9.81E-11

AREG -3.560067675 4.02E-12

HAS2 -3.560397891 2.96E-10

SAMD9 -3.568024419 5.62E-09

MBNL3 -3.568480092 5.62E-09

LCP1 -3.570078666 9.59E-13

RBM47 -3.581066616 1.28E-10

FBP1 -3.590556614 6.52E-11

RAB25 -3.596830237 7.20E-12

S100A9 -3.622500287 6.62E-11

GUCY1A3 -3.623714839 1.52E-11

LIMCH1 -3.633217488 7.25E-11

TSTD1 -3.638737699 1.58E-10

TGFB2 -3.644536202 7.71E-10

IL6 -3.671918483 1.89E-12

NPNT -3.67624806 6.44E-12

ATF3 -3.715646467 6.13E-12

CDH1 -3.72803424 1.73E-12

LOC101928916 /// NNMT -3.770920152 2.13E-10

KLK5 -3.782187031 2.14E-11

PROM2 -3.796909996 9.63E-10

CAMK2N1 -3.823699366 1.31E-12

HAS2 -3.84586045 5.69E-12

TXNIP -3.870529236 8.11E-11

JDP2 -3.871186809 4.46E-09

THBS1 -3.87718008 9.85E-12

CST6 -3.899655242 2.47E-11

TNFSF15 -3.915484882 3.41E-10

MACC1 -3.926300614 1.88E-11

SLC1A6 -4.001471651 4.02E-11

TGFB2 -4.040168387 6.44E-12

INPP4B -4.06495484 1.00E-11

IGFBP5 -4.066952799 1.17E-09

TNFSF15 -4.077822661 1.15E-09

CLDN7 -4.119386859 7.45E-10

ST14 -4.135475087 1.46E-11

GPR87 -4.158359507 1.90E-11

IFI16 -4.177072024 4.32E-12

IFI16 -4.190969472 6.87E-10

TXNIP -4.226433894 9.21E-11

EFEMP1 -4.232255636 7.83E-13

CYP1B1 -4.252470262 9.45E-11

PRRG4 -4.25421496 9.85E-12

TXNIP -4.254542223 3.63E-12

LCN2 -4.267429273 1.34E-11

JPH1 -4.313250955 1.94E-12

VCAN -4.327036146 5.77E-12

S100A14 -4.341199274 9.85E-12

CYP1B1 -4.368466035 9.85E-12

KRTAP2-3 /// KRTAP2-4 -4.369825747 2.05E-12

IFI27 -4.379013546 8.81E-10

VCAN -4.394411051 7.40E-13

RBM47 -4.441137784 2.14E-11

LINC01133 -4.497581401 2.78E-11

METTL7A -4.518041249 1.67E-11

ITGB6 /// LOC100505984 -4.520050533 3.48E-13

TGFB2 -4.549482642 1.12E-12

LIMCH1 -4.666596225 5.68E-11

GRHL2 -4.682034028 7.45E-13

WDR72 -4.780565994 3.49E-12

LOC101928916 /// NNMT -4.858537802 3.57E-11

RUNX2 -4.863994803 1.58E-10

LXN -4.926653101 9.85E-12

RGS7 -5.056754392 1.34E-11

C15orf48 -5.071046585 3.09E-13

TRBC1 -5.078199846 1.06E-13

MPZL2 -5.137913545 1.35E-11

HS3ST3A1 -5.194975978 3.33E-13

SLC1A6 -5.522947736 6.47E-12

VGLL1 -5.729182535 3.93E-13

LIMCH1 -5.852609722 2.57E-13

ESRP1 -5.943057744 9.59E-13

GJB2 -6.174483674 5.32E-13

EFEMP1 -6.195107896 2.10E-12

TACSTD2 -6.26307819 1.80E-12

LOC101928687 -6.291738981 3.93E-13

TCN1 -6.477479707 1.89E-12

ESRP1 -6.666515317 7.65E-13
